# *De novo* transcriptome sequencing and gene co-expression reveal a genomic basis for drought sensitivity and evidence of a rapid local adaptation on Atlas cedar (*Cedrus atlantica*)

**DOI:** 10.3389/fpls.2023.1116863

**Published:** 2023-04-19

**Authors:** Irene Cobo-Simón, Jèssica Gómez-Garrido, Anna Esteve-Codina, Marc Dabad, Tyler Alioto, Julin N. Maloof, Belén Méndez-Cea, José Ignacio Seco, Juan Carlos Linares, Francisco Javier Gallego

**Affiliations:** ^1^ Department of Physical, Chemical and Natural Systems. University Pablo de Olavide, Seville, Spain; ^2^ Department of Genetics, Physiology and Microbiology, Genetics Unit. Faculty of Biological Sciences, Complutense University of Madrid, Madrid, Spain; ^3^ Nacional Center for Genomic Analysis-Center for Genomic Regulation (CNAG-CRG), Centre for Genomic Regulation, Barcelona Institute of Science and Technology, Barcelona, Spain; ^4^ Department of Plant Biology, University of California at Davis, Davis, CA, United States

**Keywords:** RNA-Seq, Atlas cedar, drought sensitiveness, eco-physiology, adaptive capacity, conifers, climate change, phenotypic diversity

## Abstract

**Introduction:**

Understanding the adaptive capacity to current climate change of drought-sensitive tree species is mandatory, given their limited prospect of migration and adaptation as long-lived, sessile organisms. Knowledge about the molecular and eco-physiological mechanisms that control drought resilience is thus key, since water shortage appears as one of the main abiotic factors threatening forests ecosystems. However, our current background is scarce, especially in conifers, due to their huge and complex genomes.

**Methods:**

Here we investigated the eco-physiological and transcriptomic basis of drought response of the climate change-threatened conifer *Cedrus atlantica*. We studied *C. atlantica* seedlings from two locations with contrasting drought conditions to investigate a local adaptation. Seedlings were subjected to experimental drought conditions, and were monitored at immediate (24 hours) and extended (20 days) times. In addition, post-drought recovery was investigated, depicting two contrasting responses in both locations (drought resilient and non-resilient). Single nucleotide polymorphisms (SNPs) were also studied to characterize the genomic basis of drought resilience and investigate a rapid local adaptation of *C. atlantica*.

**Results:**

*De novo* transcriptome assembly was performed for the first time in this species, providing differences in gene expression between the immediate and extended treatments, as well as among the post-drought recovery phenotypes. Weighted gene co-expression network analysis showed a regulation of stomatal closing and photosynthetic activity during the immediate drought, consistent with an isohydric dynamic. During the extended drought, growth and flavonoid biosynthesis inhibition mechanisms prevailed, probably to increase root-to-shoot ratio and to limit the energy-intensive biosynthesis of secondary metabolites. Drought sensitive individuals failed in metabolism and photosynthesis regulation under drought stress, and in limiting secondary metabolite production. Moreover, genomic differences (SNPs) were found between drought resilient and sensitive seedlings, and between the two studied locations, which were mostly related to transposable elements.

**Discussion:**

This work provides novel insights into the transcriptomic basis of drought response of *C. atlantica*, a set of candidate genes mechanistically involved in its drought sensitivity and evidence of a rapid local adaptation. Our results may help guide conservation programs for this threatened conifer, contribute to advance drought-resilience research and shed light on trees’ adaptive potential to current climate change.

## 1 Introduction

Forest ecosystems are key sources of important ecological, economic and social functions. However, the long-lived character and limited dispersal ability of trees make these complex ecosystems particularly concerning in facing major evolutionary challenges such as the current climate change (Jump and Peñuelas, 2005). Thus, knowledge of the molecular and functional mechanisms that control adaptation and resilience (Lloret et al., 2011) of trees to drought is key in the current context of a changing climate, since recurrent drought is one of the main climatic factors threatening forests, especially in the Mediterranean region (Alberto et al., 2013; Mencuccini et al., 2015). In fact, increasing severity, duration, and higher frequency of drought events as a consequence of climate change are increasing tree mortality and forest dieback worldwide (Schueler et al., 2021). Notwithstanding, this knowledge is scarce, especially in conifers, due to their huge and complex genomes (Klupczyńska and Ratajczak, 2021).

In particular, to predict future tree mortality and the carbon balance of forest ecosystems as well as to develop management strategies to help forests cope with this increasing drought occurrence, a better understanding is needed (i) of the physiological limits and traits connected to drought tolerance of individual trees (Sohn et al., 2013; Bennett et al., 2015; Trujillo-Moya et al., 2018), (ii) of the genetic diversity of traits related to drought resistance and avoidance (iii) of local adaptations and the adaptive capacity of single species (Schueler et al., 2021), for example, relicts (Hampe and Jump, 2011).

Relict species are populations left behind during climate-driven range shifts which persist in areas with benign but sub-optimal environmental conditions surrounded by an unfavorable regional climate (Hampe and Jump, 2011). Some adaptations to these sub-optimal habitats might be key to cope with the current changing climate (e.g. Kawecki, 2008; Sexton et al., 2009). Relicts usually have small population sizes, isolation and limited possibilities for migration, leading to reduced gene flow and genetic diversity, which make them unique experimental models to test the adaptive potential of trees to a changing environment (Hampe and Petit, 2005; e.g. Cobo-Simón et al., 2020; Cobo-Simón et al., 2021; Cobo-Simón et al., 2022). Despite this, some studies have found no evidence of decreasing population sizes or fitness (Hampe and Jump, 2011). Therefore, it has been hypothesized that they might retain genetic variants for some ecologically relevant traits (Kawecki, 2008).

Here we focus on *Cedrus atlantica*, a relict conifer endemic from North Africa (Algeria and Morocco), distributed in mountain ranges through altitudinal ecotones (Abel-Schaad et al., 2018). Despite its relatively wide tolerance to climate and soil type (Abel-Schaad et al., 2018), it is threatened by climate change, particularly by the projected temperature increase. Furthermore, its distribution range has undergone a dramatic reduction over the last decades due to the increasing aridity and human activities (Abel-Schaad et al., 2018). Its presence in Spain is product of a reforestation project carried out in southern Iberian Peninsula during the last century from natural stands of northern Morocco. This reforestation project further highlights *C. atlantica* wide tolerance to varied environmental conditions, since populations located at different regions, with remarkable differences in humidity and precipitation, do not show apparent differences in fitness. Hence, this reforestation project offers a natural experiment to investigate the molecular and functional mechanisms of adaptation to drought in conifers. To the best of our knowledge, it has never been performed in conifers.

The objective of this work is to investigate a possible rapid local adaptation to drought of *C. atlantica* populations at Southern Spain. Moreover, we aim to investigate the molecular and functional basis of drought resilience in this climate change-threatened conifer. To this end, we study the mechanistic connection between gene expression/eco-physiology and drought resilience in two *C. atlantica* populations located in areas with contrasting differences in precipitation: Fiñana (Almería), characterized by strong droughts during the summer, and Dornajo (Granada), with higher precipitation rate. First, *C. atlantica* seedlings from both locations were subjected to controlled drought conditions, based on our field data (Linares et al., 2012; Sanchez-Salguero et al., 2015). Then, we investigated the differences in gene expression through the immediate (24 hours) and extended (20 days) drought responses and between two contrasting post-drought recovery phenotypes (resilient and sensitive) *via* RNA-seq within and between both studied populations. Single nucleotide polymorphisms (SNPs) between these contrasting phenotypes and the two locations were also analyzed to characterize their genomic basis and to investigate a rapid local adaptation. During the drought treatments and the post-drought recovery, two eco-physiological variables related to drought response were tracked (net photosynthesis and stomatal conductance) to investigate differences in the eco-physiological response to drought and post-drought recovery between locations.

We hypothesize a rapid local adaptation of the studied *C. atlantica* populations to the contrasting precipitation rates of both locations, supported by physiological, gene expression and genomic differences (SNPs). Furthermore, we hypothesize that contrasting gene expression is related to carbon and water balance variability in response to drought stress and causes the two contrasting post-drought recovery phenotypes in both locations. Given that resilient and sensitive individuals from both locations were subjected to the same drought treatment (extended), we expect SNPs as polymorphisms associated to their differentially expressed genes, and thus, related to their drought resilience. Although other molecular mechanisms might be acting (e.g. epigenetic regulation), their characterization is beyond the scope of this study.

## 2 Materials and methods

### 2.1 Plant material, growth conditions and drought stress experiment

**Figure 1:**
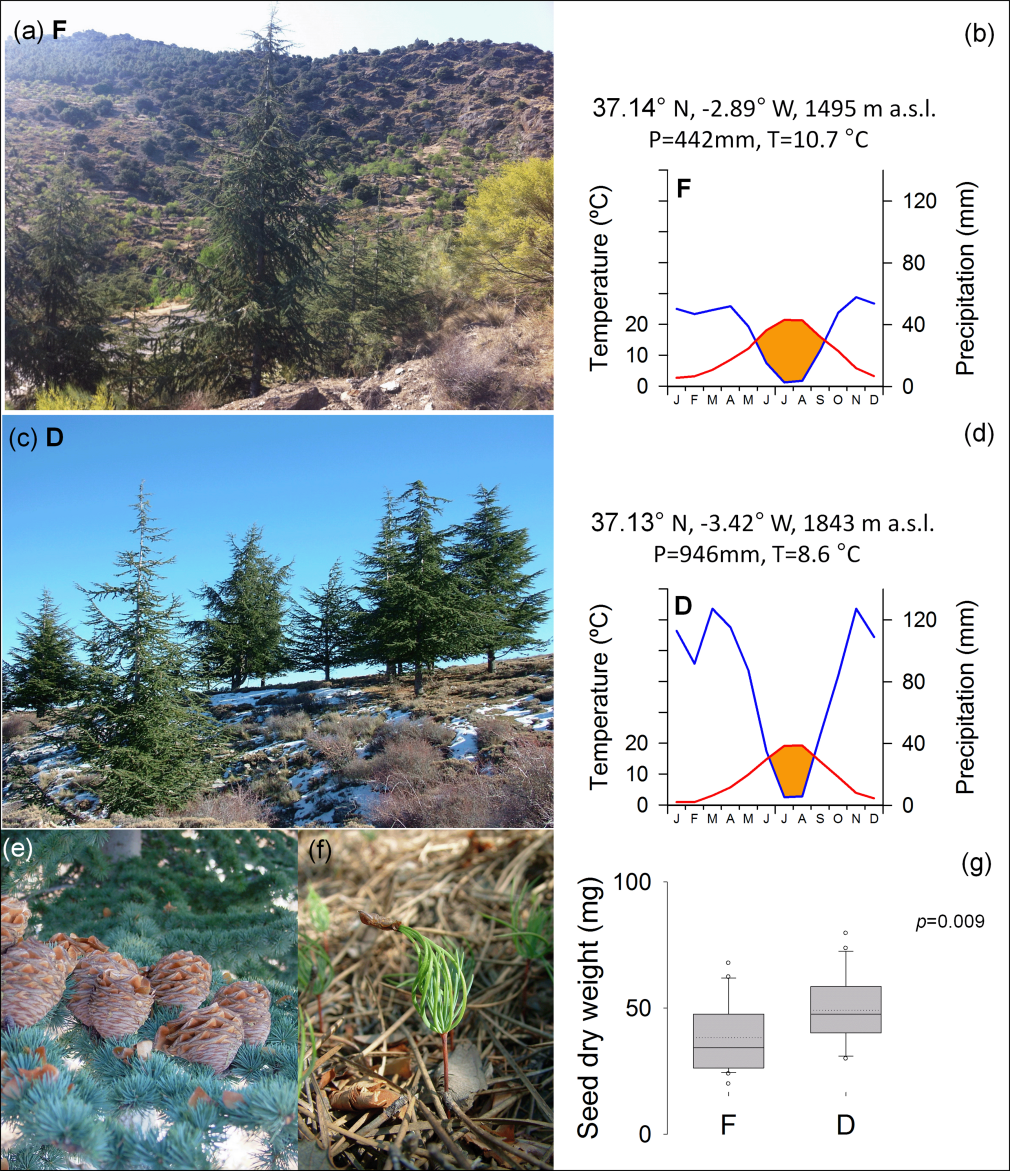
Atlas cedar (*Cedrus atlantica*) trees at Fiñana, F **(A)** and Dornajo, D **(C)** sites (Sierra Nevada range, south Spain). Sampling location (latitude N, longitude W, and elevation m a.s.l.) and mean climate for the period 1971-2021 are indicated for F **(B)** and D **(D)** sites, respectively. The blue lines indicate the monthly total precipitation, the red lines indicate the monthly mean temperature, the shaded orange area indicates the water deficit period; total annual precipitation (P, mm) and mean annual temperature (T, °C) are also noted. Mature cones with spreading seeds **(E)** and germinating seedlings **(F)** were highly viable in both sites. Mean seed dry weight **(G)**; the inset indicates the p value for ANOVA.

A drought stress experiment was carried out using Atlas cedar seedlings germinated from seeds sampled in two afforested populations of southern Spain (Fiñana, F and Dornajo, D, respectively; **Figures 1A, C**). The reforestation project was performed with *C. atlantica* individuals from natural stands of northern Morocco during the last century. The study sites show contrasting climate conditions due to a longitudinal gradient, where aridity increases eastward, and due to elevation, where aridity increases downward. F site (Almería), is characterized by higher mean temperature and lower total precipitation, which determined increased summer drought, compared to D site (**Figures 1B, D**). During the period 2011-2015, air temperature (T) was registered hourly in both locations using a Hobo H8 data logger. Data loggers were placed inside open-bottom PVC cylinders situated at 1.5m from the ground and covered by sealing foam to prevent direct heating from the sun. Further, to obtain long-term climate data, monthly total precipitation (P) and T were downloaded from the EOBS database v23.1e for the period 1971–2021 (Haylock et al., 2008) using the Climate Explorer webpage (https://climexp.knmi.nl/). Local data of monthly total precipitation were also used to account for elevation gradients.

Seeds were obtained in the field from mature cones (**Figure 1E**) and germinated (**Figure 1F**) in the common garden of the University Pablo de Olavide (Seville, Spain). Both populations showed similar germination percentage (80.1% and 82.1%, for F and D, respectively), while seed dry weight was significantly higher in D site (**Figure 1G**). Seedlings grown in 0.77-liter pots with 38.5 cm^2^ of surface exposed to evaporation, filled with sterile sand and perlite substrate in 3:1 ratio for about one year in the common garden. Environmental conditions in the common garden (temperature, relative humidity and PAR) were monitored using Onset Computing HOBO Pro v2 data loggers (HOBO U23-series). Mean annual air temperature was 17.9°C, 27.4°C in the hottest month (August), and 10.3°C in the coldest month, (January). Mean annual relative humidity was 68.3%, 55.9% in the drier month (July), and 81.2% in the wettest month (November). Mean PAR was 445.8 lux. Irrigation was provided to saturation. The seedlings were fertilized in pots with solid NPK synthetic fertilizer, containing 12.0% of total Nitrogen (N), of which 6.5% was nitric Nitrogen (N-NO_3_), and 5.5% was ammoniacal Nitrogen (N-NH_4_); Phosphorus pentoxide (P_2_O_5_) soluble in neutral ammonium citrate and water 12.0%, of which 6.0% was Phosphorus pentoxide (P_2_O_5_) water soluble; Sulfur trioxide (SO_3_) total 15.0%, of which 12.0% was Sulfur trioxide (SO_3_) water soluble; Magnesium Oxide (MgO) Total 2.0%, of which 1.5% was Magnesium oxide (MgO) water soluble; Potassium oxide (K_2_O) water soluble 17.0%, Boron (B) total 0.02%, and Zinc (Zn) total 0.01%. Then, a subset of seedlings were randomly selected and introduced in controlled growth chambers (CGC) under constant conditions of humidity (70% relative humidity RH) and temperature (27°C). CGC air vapour pressure deficit (VPD, kPa) was calculated using temperature and relative humidity in the following equations (Sanchez-Salguero et al., 2015):

Saturated vapour pressure (svp) was calculated as: $svp=0.66*e^{\left(0.06*T\right)}$

Then, VPD was obtained as: $VPD=\left(\frac{\left(100-RH\right)}{100}\right)*svp$ where RH is the CGC air relative moisture (%), and T is the CGC air temperature (°C).

Independent of the following drought stress treatment, all seedlings stayed 15 days in CGC for acclimation, until the variables used to monitor control seedlings hydric status (net photosynthesis and stomatal conductance) showed high and stable values. Seedlings (both controls and those subjected to drought treatments) were monitored weekly by means of a gas exchange system provided with an infrared gas analyzer (IRGA) (LI-COR, LI-6400XT, EEUU). During this acclimation period, watering was carried out by adding 10 mL of water per day. After the acclimation, the variables used to monitor control seedlings (A and Gs) were relatively steady and averaged (± standard error) (A = 6.5 ± 0.5 μmol CO2·m^-2^·s^-1^, Gs = 110 ± 13 mmol H_2_O cm^-2^ s^-1^). Photoperiod was stablished between 7:00 AM and 19:00 PM (12 hours light/12 hours darkness, light at 600 lux), while physiological measurements and further sampling for RNA extraction were performed between 10:00 AM and 1:00 PM, again, for both controls and seedlings subjected to the drought treatments.

**Figure 2:**
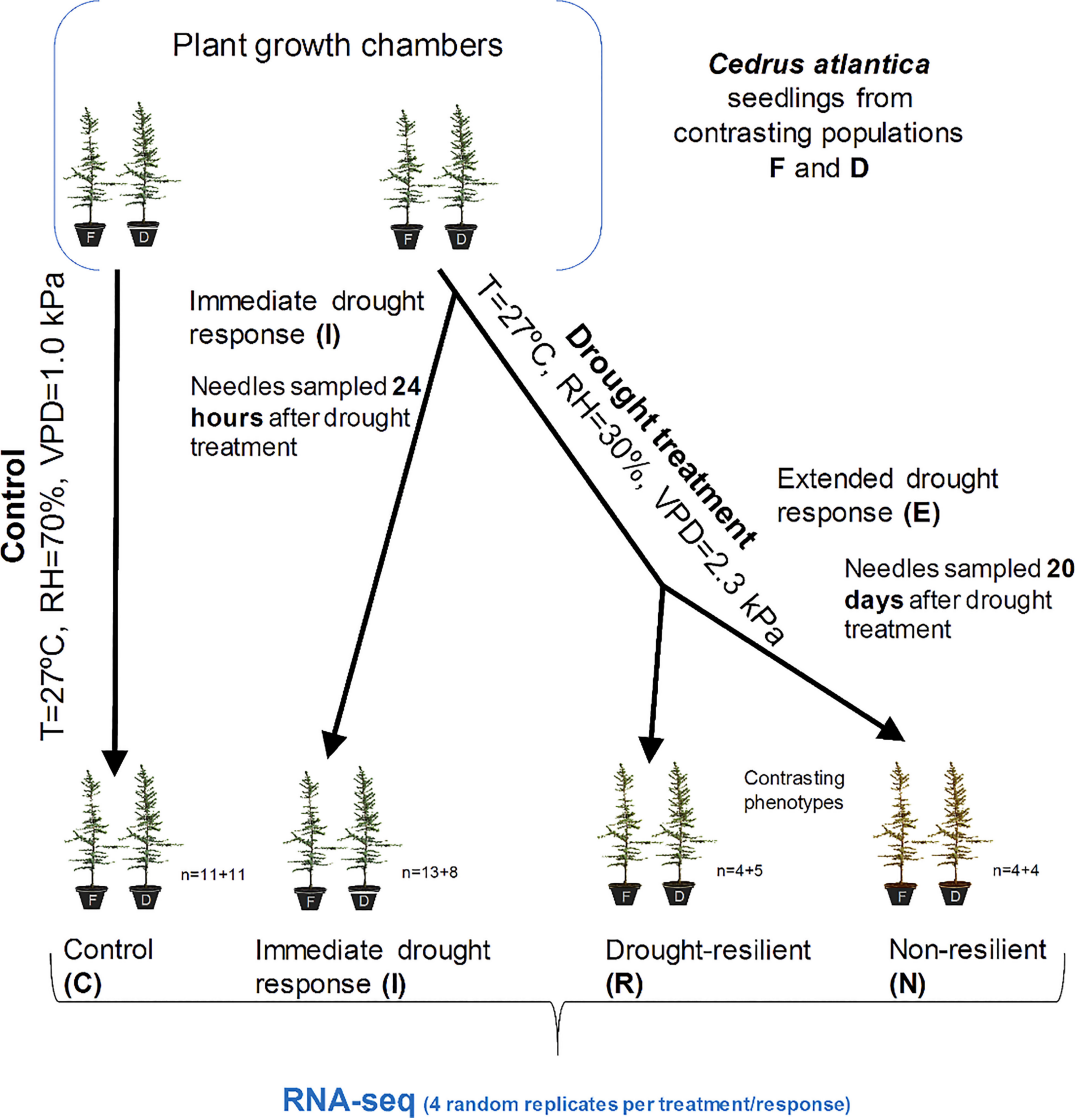
Drought treatment experimental design and sampling procedure. Net photosynthesis (A) and stomatal conductance (Gs) were monitored in controls (C), immediate drought (**I**, after 24 hours of drought treatment), and extended drought (E, after 20 days of drought treatment).The E group was divided into drought resilient seedlings (R) and non-resilient seedlings (N), based on recovery capacity after the extended drought. T, temperature; RH, relative humidity; VPD, vapor-pressure deficit; n indicates the number of individuals from Fiñana (F) and Dornajo (D), respectively (n=F+D).

Drought treatment (**Figure 2**) was applied to 38 seedlings by reducing the RH of the CGC up to 30%, which is equivalent to a VPD of 2.3 kPa, whereas 22 remained as control (RH=70%), which is equivalent to a VPD of 1.0 kPa. Both CGC maintained 27 ° C of temperature and 10 mL per day of watering. Immediate drought response, extended drought response and drought resilience were assessed using gas exchange monitoring and gene expression by RNA extraction (**Figure 2**). This experimental design allows a quantification of drought effects at a molecular resolution for seedling subjected to experimental drought. Drought resilience was defined here as the capacity of a seedling to reach gas exchange rates (A and Gs) similar to those prior to drought treatment. Defined this way, drought resilience encompasses the capacity to cope with drought, that is, resistance, and the ability to return to prior gas exchange levels after drought, that is recovery (Lloret et al., 2011). We defined three sample types within the experiment (**Figure 2**): (i) Controls (C, n=11 from Dornajo and n=11 from Fiñana), seedlings were raised in CGC at 70% RH; (ii) immediate drought response treatment (I, n=8 from Dornajo and n=13 from Fiñana), seedlings were raised in CGC at 30% RH and needles for RNA extraction were collected 24 hours after; and (iii) extended drought response treatment (E, n=9 from Dornajo and n=8 from Fiñana), seedlings were raised in CGC at 30% RH and needles were collected for RNA extraction 20 days after the drought treatment started (**Figure 2**).

The recovering drought response treatment introduces the concept of drought resilience (Lloret et al., 2011). E treatment seedlings were returned to control conditions after 20 days of drought treatment (**Figure 2**). Then, recovery (regarding A and Gs values observed after drought) was monitored to quantify drought resilience (*sensu* Lloret et al., 2011). According to that, we defined two groups: drought resilient seedlings (R, n=4 from Fiñana; n=5 from Dornajo), defined as those E seedlings that reached, at least, the 30% of their A and Gs values in control conditions in less than 3 days; and non-resilient seedlings (N, n=4 from Fiñana, n=5 from Dornajo), defined as those E seedlings that died or did not manage to reach, at least, the 30% of their A and Gs values in control conditions in less than 3 days (**Figure 2**). We set this threshold (30%) based on our own experience on previous essays, where we determined that, overall, seedlings that are able to recover about 1/3 of their initial rates are thereafter able to survive after the drought conditions end. All collected needles were stored in a freezer at -80°C until RNA extraction was performed.

### 2.2 RNA extraction, cDNA library preparation and RNA-sequencing for transcriptome analysis

Four individuals belonging to each treatment/response (C, I, R, N) from each location (Fiñana and Dornajo) were selected for the RNA extraction to analyze the same quantity of biological replicates. Spectrum Plant Total RNA Kit (Sigma-Aldrich, Saint Louis, MO, USA) was used to carry out a total RNA extraction from the collected needles. The manufacturer’s protocol was followed with one modification. 2% polyvinylpyrrolidone (PVP) K30 (Sigma-Aldrich) was added to the lysis buffer to enhance the extraction efficiency. Bioanalyzer 2100 (Agilent Technologies, Inc, Santa Clara, CA, USA) was used to test the quality of the extracted RNA. A ND-1000 Spectrophotometer (NanoDrop™, Thermo Fisher Scientific, Waltham, MA, USA) was used to measure its concentration. 1.8 ≤ OD260/280 ≤ 2.2, OD260/230 ≥ 1.8 and RNA Integrity Number (RIN) ≥ 7 were applied as quality standards. Then, all samples were sent to the National Center for Genomic Regulation (CNAG-CRG, Barcelona, Spain) for sequencing. There, quality control (QC) was performed by fluorescent-based quantification (RiboGreen, Invitrogen™, Thermo Fisher Scientific) and integrity evaluation (2100 Bioanalyzer, Agilent Technologies, Santa Clara, CA, USA), followed by a cDNA library preparation (Illumina Stranded mRNA Prep) and RNA-sequencing by Illumina HiSeq2500 (Illumina, Inc, San Diego, CA, USA).

### 2.3 Bioinformatic analysis

#### 2.3.1 *De novo* transcriptome sequencing and functional annotation

*De novo* transcriptome assemblies were performed for each treatment/response (C, I, R, N) in both studied populations (Fiñana and Dornajo) using Trinity (Grabherr et al., 2011) v2.8.4 and Bridger (Chang et al., 2015) version r2014-12-01, after performing read trimming and normalization using Trimmomatic (version 0.36, Bolger et al., 2014) and Trinity, respectively. Assembly quality for the obtained transcriptomes was compared in terms of contiguity (N50) and completeness with BUSCO (Simão et al., 2015) v3.0.2 using a plant specific dataset made of 956 genes. Finally, the Bridger approach was selected as the best one and RapClust (Trapnell et al., 2013) v0.1 was run on each of the Bridger assemblies in order to reduce redundancy before merging the four resulting transcriptomes together again with RapClust. In this process, a pseudoalignment is first performed with Sailfish (Li et al., 2010) v0.10.0 and then RapClust is used to cluster the assembled sequences into contained isoforms, in order to reduce redundancy and to cluster together all the isoforms that are likely to belong to the same gene. After obtaining the reference transcriptome, Open Reading Frames were annotated in the assembled transcripts with TransDecoder (Haas et al., 2013) v5.5.0 and functional annotation was performed on the annotated proteins with Blast2GO (Conesa et al., 2005) v 3.2. First, a BlastP (Altschul et al., 1990) search was made against the nr database from NCBI (last accessed January 2019) and InterProScan (Jones et al., 2014) 5.32-71.0 was run to detect protein domains on the annotated sequences. All these data were combined by Blast2GO which produced the final functional annotation results.

#### 2.3.2 Atlas cedar RNA-seq data sets

Raw reads for each individual were trimmed in order to remove low quality reads (mean quality score<15; reads length<36) using Trimmomatic (version 0.36, Bolger et al., 2014). Then, FastQC (version 0.11.7, Andrews, 2014) was used to test read quality. Overrepresented sequences (adapters) were removed from all samples with Trimmomatic since they were abundant in all FastQC reports. The filtered reads quality was again tested using FastQC. The read from each pair-end showing the highest quality was selected to develop the following differential expression (DE) analysis based on single-end reads. Kallisto (version 0.44.0, Bray et al., 2016) was used to align the selected reads against the *de novo* sequenced reference transcriptome for *C. atlantica*. Genes in the reference with more than 10 reads in 3 or more samples were retained for subsequent analysis.

#### 2.3.3 Differential expression analysis

The edgeR R package (Robinson et al., 2010; McCarthy et al., 2012) was used to perform a pairwise DE analysis at the gene level according to previously published protocols (Anders et al., 2013). TMM (Trimmed Mean of M Values) (Robinson and Oshlack, 2010) was applied as normalization method. Principal Component Analysis (PCA) between samples was performed to explore the data structure. DE analysis was conducted as follows: I versus C, using C as baseline, to find genes implied in the immediate drought response; E versus I, using I as baseline to obtain genes implied in the extended drought response; and R vs N, using N as baseline, to obtain potential candidate genes of drought resilience. The cut-off criteria of a false discovery rate (FDR)-adjusted P-value ≤ 0.05 was used to consider differentially expressed genes (DEG). Log fold change threshold = 0 was used to calculate up and down-regulated genes.

#### 2.3.4 Gene co-expression network analysis

Gene co-expression network analysis was performed to cluster the reads into modules of highly co-expressed genes based on their expression similarity using the R package WGCNA (Langfelder and Horvath, 2008; Langfelder and Horvath, 2012). First, read alignments were normalized using “voom” fuction in limma R package (Bioconductor release 3.4, Law et al., 2014). Sample clustering was carried out to identify possible outliers. Genes were represented by nodes in the gene co-expression network. The similarity between expression profiles of paired genes was obtained by a Pearson correlation. To construct an adjacency matrix, a power function (β) was applied on the Pearson correlation matrix. To balance the scale-free property of the co-expression network and the sparsity of connections between genes, a value of 14 was given to the β. A signed co-expression network was constructed, in which modules correspond to positively correlated genes, since it is more accurate for this work. The obtained adjacency matrix was used to build a topographical overlap matrix (TOM). A hierarchical clustering was developed in order to group the genes based on dissimilarity of gene connectivity, defined as 1–TOM. Co-expression clusters were calculated by implementing the cutreeDynamic function with the following parameters: deepSplit = 2, pamRespectDendro = F, minClusterSize = 30. Finally, highly correlated clusters were merged to obtain modules in the network using mergeCloseModules function with a cutHeight set to 0.25 (see WGCNA code at Appendix 2).

An overlapping analysis between the genes belonging to the obtained modules and the DEGs was performed to select the most meaningful modules for our study. The DEGs were separated into up and down-regulated beforehand. The statistical significance of the overlap was calculated by Fisher’s exact test. Modules with a statistically significant p-value (P<0.05) were selected for further analysis.

An expression-value heatmap was developed to cluster and visualize the log2 fold-change for gene expression of each selected module using the R package *pheatmap* (version 1, Kolde, 2012). Modules with the expected expression pattern, considering our experiment, were selected. We focused on highly connected genes or hub genes per module because they are probably crucial components of the network, given their central location (Miao et al., 2017). Hub genes were identified by visualizing the modules and selecting the most interconnected genes in each module using Cytoscape version 3.9.1 (Shannon et al., 2003). The GO-seq package (Young et al., 2010) in R was used to perform GO term enrichment analysis to functionally annotate the hub genes and the selected modules for testing whether their functions were biologically meaningful. Only those GO terms with FDR-adjusted P-value< 0.05 were considered statistically significant. The GO term enrichment results were visualized using REVIGO (Supek et al., 2011).

#### 2.3.5 Single nucleotide polymorphisms (SNP) calling

To investigate the existence of genetic variations (SNPs) associated with the differences in gene expression between the two post-drought phenotypes (R and N) and locations (Fiñana and Dornajo), variant detection was performed. This analysis was conducted to support the DE analysis and WGCNA results, since the low number of biological replicates was not enough to draw statistically robust conclusions. Paired-end reads were aligned with BWA program (version 0.7.17, Li and Durbin, 2009). Picard (version 2.9.2) was used to sort the alignments in genome order and to mark the pseudo-duplicates of DNA fragments which can occur during library preparation. Finally, Freebayes (version 1.3.4, Garrison and Marth, 2012) was used to call SNPs against the reference transcriptome. The resulting SNPs were plotted in a Principal Components Analysis (PCA). We filtered the SNP list to keep only those SNPs with fixed differences between all R and N samples, that is, those which are homozygous for one allele in all R samples and homozygous for a different allele in all N samples. To this end, we used BCFTOOLS (version 1.16, Li, 2011). We also filtered the SNP list to keep fixed SNPs between all Fiñana and Dornajo samples. Then, the GO-seq package (Young et al., 2010) in R was used to perform GO term enrichment analysis to functionally annotate the fixed SNPs between locations and responses to test whether their functions were biologically meaningful. Only those GO terms with FDR-adjusted P-value< 0.05 were considered statistically significant. The GO term enrichment results were visualized using REVIGO (Supek et al., 2011).

### 2.4 RNA validation with real-time quantitative pcr (qRT-PCR) of RNA-seq results

To confirm the results from the RNA-seq analysis, some DEGs were selected, and their expression levels were confirmed by qRT-PCR analysis. For the quantitative real-time PCR (qRT-PCR), a TB Green Advantage qPCR Premix (Takara, Japan) was used and run on a QuantStudio 7 Flex Real-Time PCR System (Thermofisher, USA). For each gene, the measurements were performed in four replicates, and the average cycle thresholds (Ct) were used to determine the fold-change. The expression ratio (Log2FC) change was calculated by the 2-ΔΔCT method. 18S rRNA gene was used as a reference gene to normalize the qRT-PCR data. All of the primers used for the qRT-PCR are listed in **Table S1**.

## 3 Results

### 3.1 Drought stress experiment

**Figure 3:**
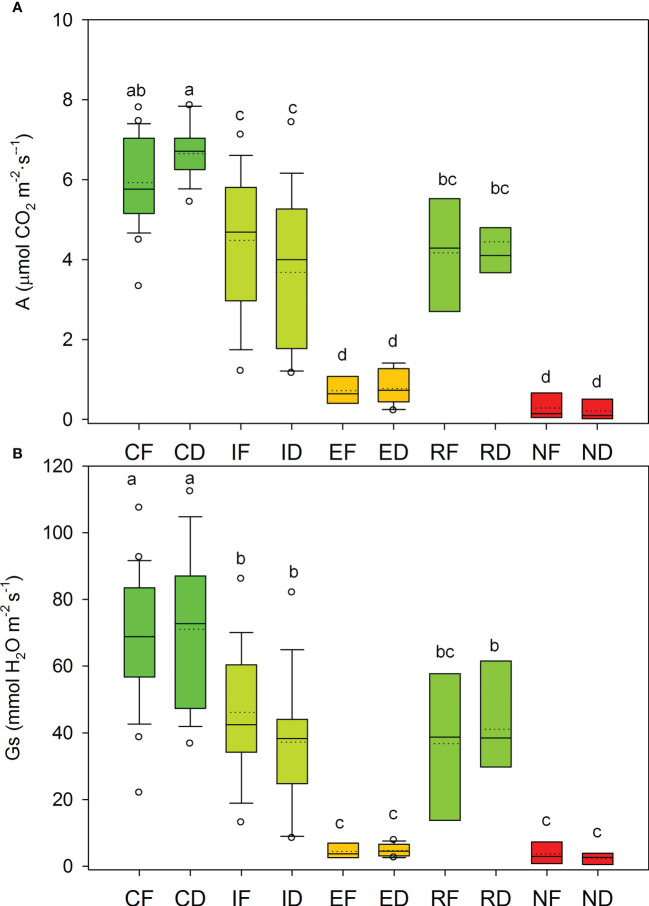
Net photosynthesis, A **(A)** and stomatal conductance, Gs **(B)** measured in seedlings from Fiñana (F) and Dornajo (D) subjected to treatments: control (C), immediate drought (I, after 24 hours of drought treatment), and extended drought (E, after 20 days of drought treatment).The E group was divided into drought resilient seedlings (R) and non-resilient seedlings (N), based on recovery capacity after the extended drought. Groups are noted by the treatment/response (C, I, E; R, N) and population codes (F, D). Different letters indicate significant diferenteces (p<0.05) by ANOVA.

Net photosynthesis (A) and stomatal conductance (Gs) showed non-significant differences between populations, while drought treatment yielded a significant gas exchange decline (**Figure 3**; Supplementary Material, **Table S2**). During the post-drought recovery, R individuals showed significantly higher values of A and Gs, compared to N individuals, in both populations (**Figure 3**, **Table S2**; see ANOVA results at Appendix 1).

### 3.2 Bioinformatic analysis

#### 3.2.1 *De novo* transcriptome sequencing

The final assembly was made of 452,220 assembled contigs with a contig N50 of 1835bp. The transcripts were clustered in a total of 115,334 genes, 89,137 of which were protein-coding. According to BUSCO, our reference transcriptome had 94.3% of complete genes, 1.2% fragmented genes and only a 4.5% of missing genes out of the 452,220 assembled contigs. Taking into account that not all the genes would be present in our samples, due to lack of expression (~80% of the genes of an organism are expected to be expressed), we can conclude that we obtained the complete transcriptome for our cases. 200,028 out of the 452,220 transcripts were annotated as protein coding, of which 196,133 (98%) were functionally annotated with protein descriptions and 172,977 (86%) with GO terms.

#### 3.2.2 Atlas cedar RNA-seq data sets

Four biological replicates from each of the four treatments/responses (C, I, R and N) and from the two analyzed populations Dornajo (Granada) and Fiñana (Almería) were selected for RNA extraction (32 samples). However, a total of 17 RNA-seq libraries were successfully sequenced, 12 belonging to Fiñana and five belonging to Dornajo, since the RNA extraction was not successful in 11 individuals from Dornajo and four individuals from Fiñana. Thus, four RNA-seq libraries from C and I treatments, and two from R and N responses (each) were successfully sequenced for Fiñana samples. Two RNA-seq libraries from C, and one RNA-seq library from I, R and N treatments/responses (each) were successfully sequenced for Dornajo (**Table 1**).

**Table 1:** Differentially expressed genes between treatments/responses (FDR<0.05).

	I vs C	E vs I	R vs N
Fiñana (12 samples)			
Up-regulated	78 (0.093%)	3073 (3.6%)	3037 (3.6%)
Not significant	84,053	79,660	75,197
Down-regulated	181 (0.21%)	1579 (1.9%)	6078 (7.2%)
Fiñana + Dornajo (17 samples)			
Up-regulated	144 (0.16%)	5807 (6.4%)	6825 (7.6%)
Not significant	89,805	82,778	72,616
Down-regulated	254 (0.28%)	1618 (1.8%)	10762 (12%)

Log fold change threshold = 0 was used to calculate up and down-regulated genes. Fiñana: C = 4 samples, I=4 samples, R=2 samples, N=2 samples; Dornajo: C=2 samples, I= 1 sample, R=1 sample, N=1 sample.

17 cDNA libraries yielded approximately 528.94 million 76-bp single-end reads (**Table S3**). Quality trimming and filtration yielded 429.6 million cleaned reads that were mapped to the reference *C. atlantica de novo* transcriptome. A total of 452,220 counts were obtained after mapping reads against the reference transcriptome. After applying TMM normalization and the filtering criteria for defining an “expressed” gene (more than 10 reads in 3 or more samples) a total of 90,203 transcripts were obtained for all 17 samples.

#### 3.2.3 Differential expression analysis

**Figure 4:**
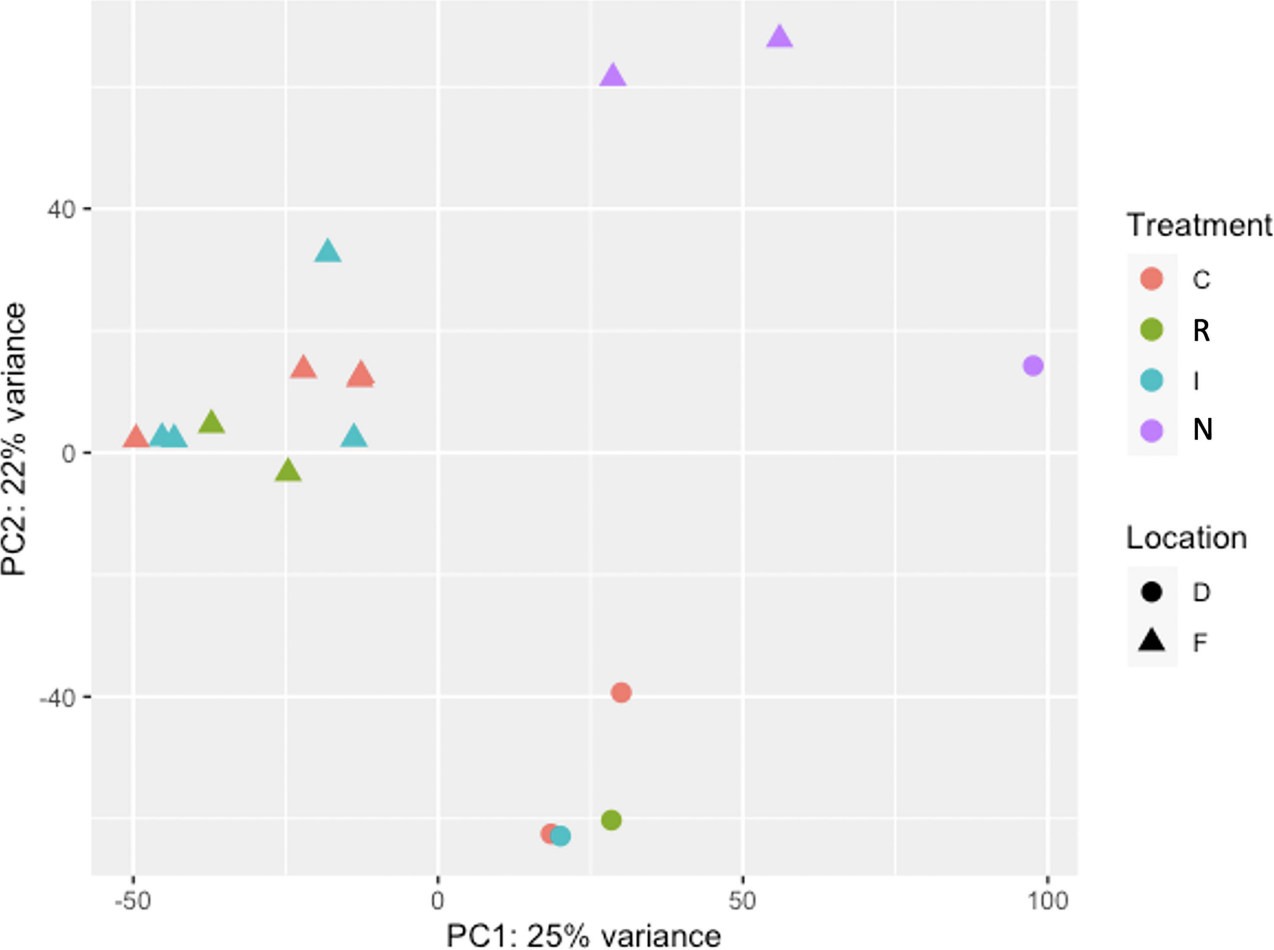
Principal Component Analysis (PCA) among the 17 normalized samples belonging to the four drought treatments/responses (Control C, immediate I, drought resilient R and non-resilient N) from Fiñana (F) and Dornajo (D) locations.

The Principal Component Analysis (PCA) between samples was plotted using a multi-dimensional scaling (**Figure 4**). The two studied locations (Fiñana and Dornajo) were clearly separated. Samples were not clustered by drought treatment (C, I, E) at any of the two locations; however, N response was clearly separated from the rest of samples in both locations. Furthermore, N individuals clustered between locations. Given the insufficient number of biological replicates per treatment/response, it was not possible to perform the DE analysis in Dornajo. Nevertheless, considering the clear separation between locations in the PCA, we decided to perform the DE analysis only in Fiñana population on the one hand, and combining both Fiñana and Dornajo populations on the other hand, in order to investigate the DEGs regardless the location. This Fiñana + Dornajo analysis is biologically meaningful since both locations were afforested with *C. atlantica* individuals from natural stands of northern Morocco. The results of both DE analyses (Fiñana and Fiñana + Dornajo) were consistent, showing the lowest number of DEGs during the I treatment, followed by E treatment, and finally, the highest number of DEGs were found between R and N responses (**Table 1**). Therefore, they showed a greater effect of the response compared to the drought treatment in the gene expression of *C. atlantica*. Nevertheless, it was remarkable that the percentage of DEGs in all three pairwise comparisons was higher when analyzing both populations together (Fiñana+Dornajo), compared to Fiñana samples (**Table 1**). To confirm the results from the RNA-seq analysis, some DEGs were selected, and their expression levels were confirmed by qRT-PCR analysis. The qRT-PCR results showed that the transcription levels of these genes were consistent with the previous RNA-seq analysis (**Figure S1**).

#### 3.2.4 Drought stress-related gene modules

**Figure 5:**
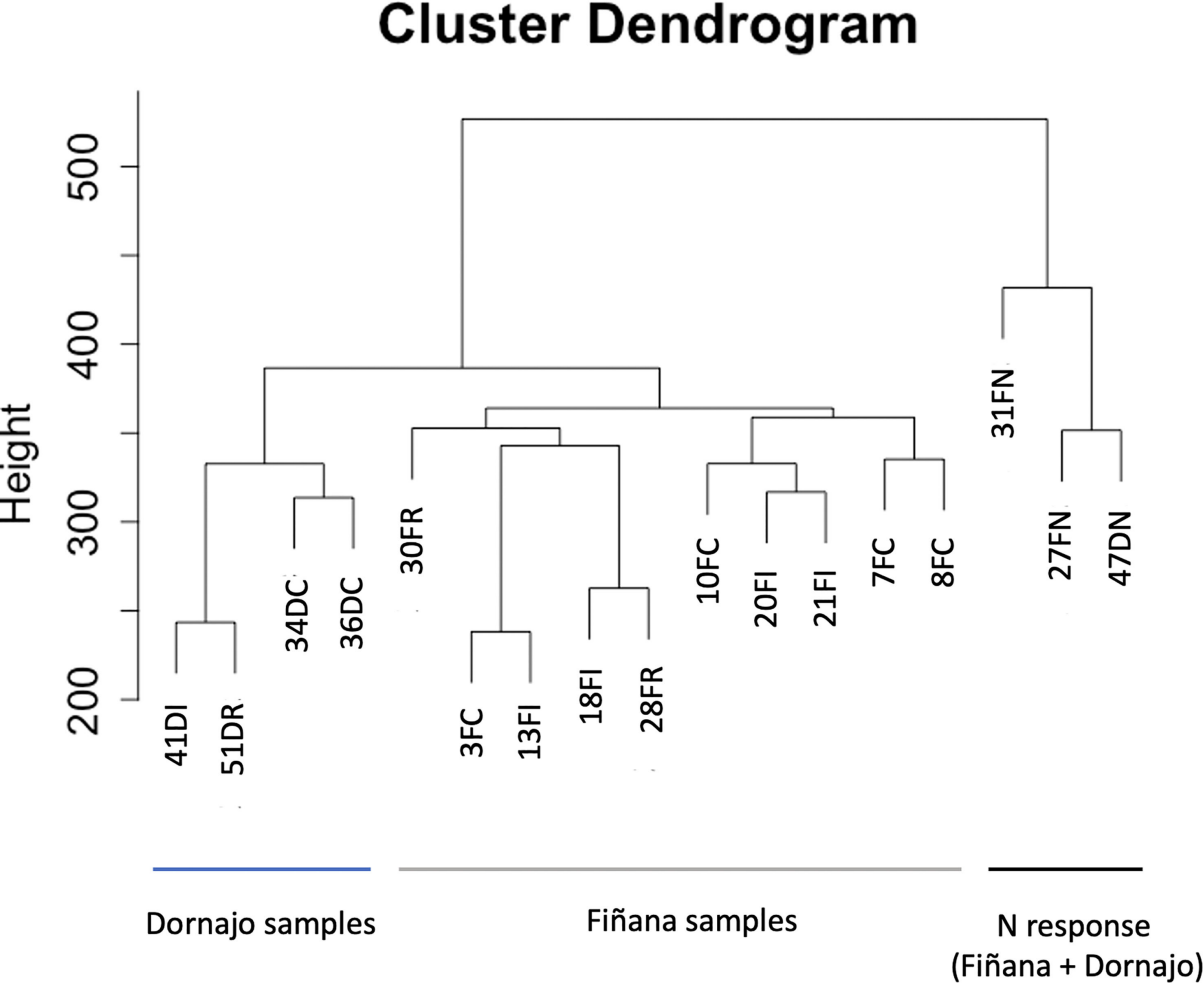
Dendrogram representing the clustering analysis of normalized samples (voom) subset for high variance with the objective of detecting outliers. Samples with a “D” after the number belong to Dornajo and with a F, to Fiñana. The last letters represent the treatment/response (C control, I immediate, R drought resilient, N non-resilient).

**Figure 6:**
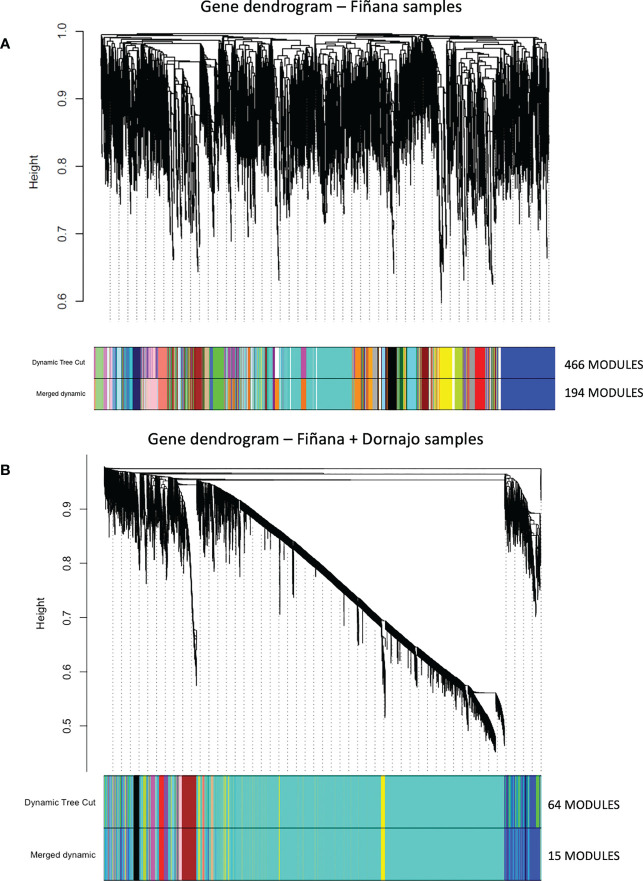
Hierarchical cluster dendrogram showing co-expression modules using weighted gene co-expression network analysis (WGCNA) of the counts to identify gene modules underlying drought stress at four different treatments and responses (C, I, R and N) in Fiñana **(A)** and Fiñana+Dornajo populations **(B)**. **(A)** 466 modules corresponding to branches are labelled with colours indicated by the colour bands underneath the tree. With 0.25 threshold merging, 194 modules were generated. **(B)** 64 modules corresponding to branches are labelled with colours indicated by the colour bands underneath the tree. With 0.25 threshold merging, 15 modules were generated.

**Figure 7:**
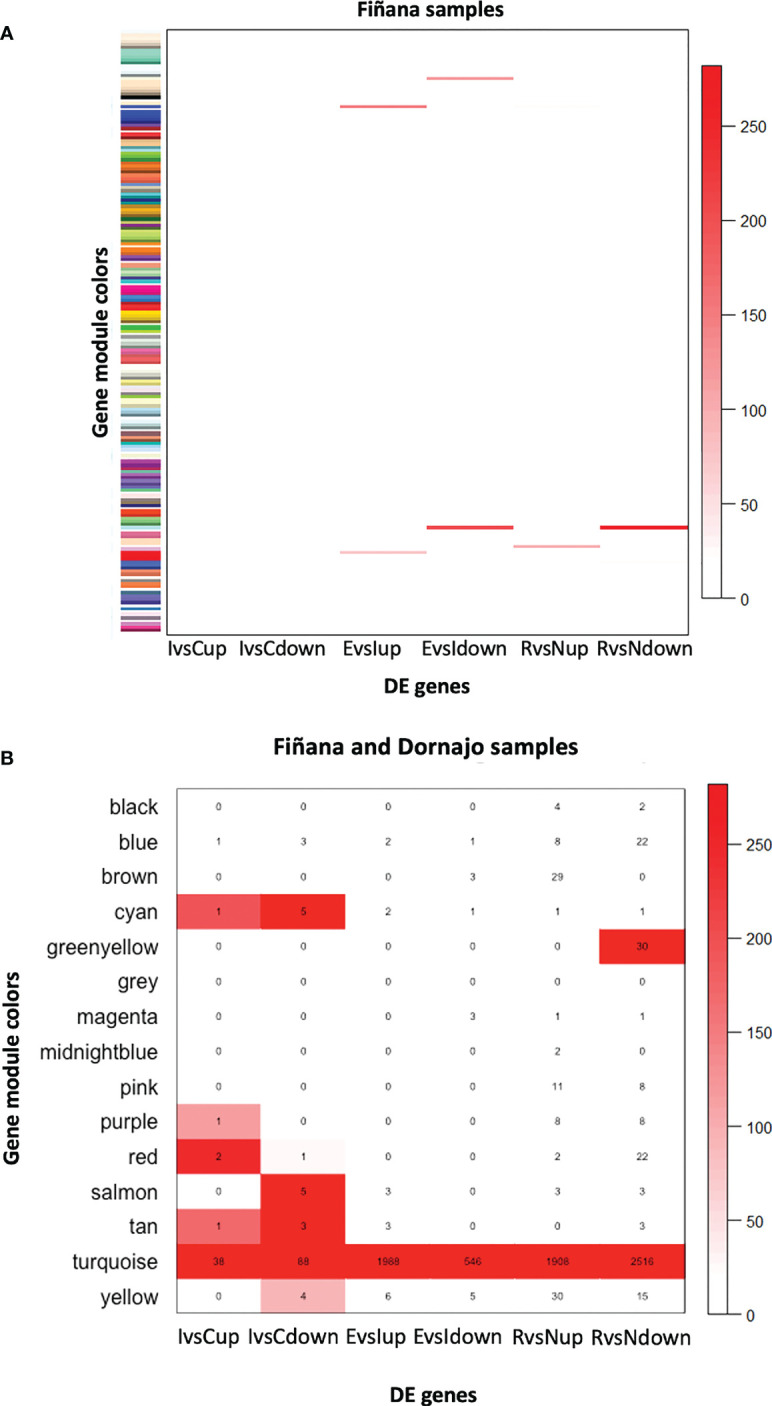
Heatmap showing the -log10(P-value) of the Fisher’s exact test calculated between the genes belonging to the 194 obtained modules in Fiñana **(A)** and to the 15 obtained modules in Fiñana + Dornajo samples **(B)**, and the pairwise DE genes up and down-regulated (IvsC up, IvsC down, EvsI up, EvsI down, RvsN up, RvsN down). Given the low number of modules in Fiñana+Dornajo, it was possible to plot the number of overlapping genes in the heatmap **(B)**. 20 modules were found to be statistically significant in Fiñana **(A)**, and 8 modules in Fiñana + Dornajo **(B)**.

**Figure 8:**
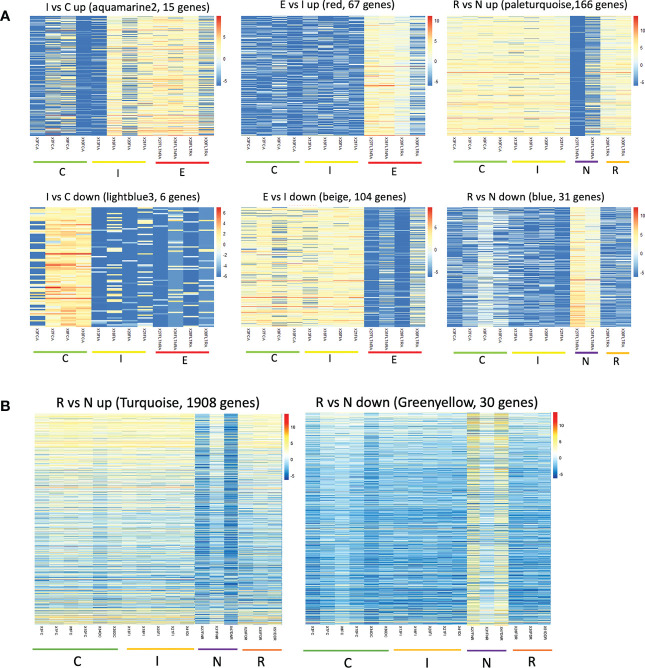
Heatmaps showing gene expression levels of the genes within the modules across three treatments (C, I, E) and responses (R, N). Warm colours represent up-regulated genes whereas cold colours represent down-regulated genes. The six modules that showed expression patterns related to the treatment/response are represented for Fiñana **(A)**, and the two modules related to the response for Fiñana + Dornajo **(B)**.

The clustering analysis did not show any outliers (**Figure 5**). Consistently with the PCA results, individuals from Fiñana and Dornajo populations were clearly separated with the exception of N individuals from both populations that clustered together. In addition, there was not clear separation among treatments/responses with the exception of N individuals from both populations. Given the high number of transcripts, genes were subset to retain the 36,359 ones with the highest variance across 12 Fiñana samples and the 12,060 ones across Fiñana + Dornajo samples (third quartile). WGCNA for Fiñana samples showed 466 modules, 194 after merging highly correlated ones (**Figure 6A**). WGCNA for Fiñana and Dornajo samples showed 64 modules, 15 after merging highly correlated ones (**Figure 6B**). To determine which modules were enriched in DEG, an overlapping table was constructed using the modules and the pairwise DEG, separately for up and down-regulated genes. 20 modules were significantly enriched in Fiñana (**Figure 7A**, **Table 2**) and 8 modules in Fiñana + Dornajo populations (**Figure 7B**, **Table 2**). Then, a heatmap was built for each significant module to select the most meaningful modules regarding their drought response pattern. Six significantly enriched modules showed an expression pattern of interest for Fiñana, related to the studied drought treatments and responses (**Figure 8A**). For Fiñana+Dornajo, two modules showed an expression pattern of interest, both related to the post-drought recovery response (**Figure 8B**). Consistently with Fiñana samples, uniquely expressed genes were found in N individuals, but not in R individuals. No modules showing an expression pattern related to the drought treatment (I or E) were found in Fiñana+Dornajo samples.

**Table 2:** Results of the enrichment analysis between pairwise DE up and down regulated genes and the 194 obtained modules in Fiñana samples, and the 15 modules in Fiñana+Dornajo samples.

Module	Pairwise comparison	Number of genes	P-value
Fiñana			
Aquamarine2	I vs C up	15	1.15 x 10^-18^
Brown	I vs C up	5	0.03
Darkseagreen1	I vs C up	6	3.63 x 10^-7^
Blue	I vs C down	4	6.69 x 10^-4^
Darkolivegreen	I vs C down	2	1.9 x 10^-3^
Lightblue3	I vs C down	6	1.15 x 10^-12^
Mediumpurple4	I vs C down	3	1.1 x 10^-4^
Blue	E vs I up	141	6.67 x 10^-147^
Darkolivegreen	E vs I up	13	1.35 x 10^-10^
Green.1	E vs I up	5	3 x 10^-4^
Mediumpurple4	E vs I up	11	1.08 x 10^-6^
Red	E vs I up	67	5.17 x 10^-75^
Slateblue1	E vs I up	4	3.6 x 10^-3^
Antiquewhite1	E vs I down	24	2.66 x 10^-14^
Beige	E vs I down	104	4.42 x 10^-118^
Darkgreen	E vs I down	10	0.01
Gold1	E vs I down	16	9.22 x 10^-13^
Ivory1	E vs I down	9	4.03 x 10^-10^
Lavenderblush4	E vs I down	15	5.75 x 10^-8^
Mediumspringgreen	E vs I down	14	8.11 x 10^-6^
Paleturquoise	E vs I down	161	4.76 x 10^-199^
Pink.1	E vs I down	22	8.52 x 10^-18^
Royalblue	E vs I down	10	0.02
Paleturquoise	R vs N up	166	2.84 x 10^-282^
Royalblue	R vs N up	30	1.62 x 10^-24^
Blue	R vs N down	31	1.09 x 10^-23^
Darkolivegreen2	R vs N down	7	1.6 x 10^-4^
Lavenderblush4	R vs N down	7	3.97 x 10^-6^
Pink.1	R vs N down	51	2.94 x 10^-95^
Fiñana+Dornajo			
Cyan	I vs C up	1	3.14 x 10^-117^
Purple	I vs C up	1	6.45 x 10^-76^
Red	I vs C up	2	1.34 x 10^-219^
Tan	I vs C up	1	2.39 x 10^-105^
Turquoise	I vs C up	38	3.28 x 10^-221^
Cyan	I vs C down	5	2.31 x 10^-232^
Red	I vs C down	1	1.42 x 10^-30^
Salmon	I vs C down	5	5.31 x 10^-245^
Tan	I vs C down	3	7.48 x 10^-230^
Turquoise	I vs C down	88	6.34 x 10^-301^
Yellow	I vs C down	4	1.04 x 10^-102^
Turquoise	E vs I up	1988	3.51 x 10^-275^
Turquoise	E vs I down	546	2.34 x 10^-250^
Turquoise	R vs N up	1908	5.68 x10^-234^
Greenyellow	R vs N down	30	2.23 x10^-220^
Turquoise	R vs N down	2516	4.50 x10^-280^

P-values were calculated by means of a Fisher’s exact test. Only statistically significant modules (P<0.05) are shown.

#### 3.2.5 Selection of hub genes and functional annotation of hub genes and modules

**Tables S4**-**S11** show the top most interconnected genes obtained using Cytoscape program (Shannon et al., 2003) for each of the six significantly enriched modules from Fiñana population (**Tables S4**-**S9**) and the two modules from Fiñana+Dornajo populations (**Tables S10**-**S11**). All genes had a log_2_ Fold-Change (logFC) value above 2 and, in the majority of cases, was around or above 4, including the DEGs between R and N individuals from Fiñana, with two biological replicates each (**Tables S8**, **S9**). Hence, it can be concluded that the hub genes were highly expressed.

**Figure 9:**
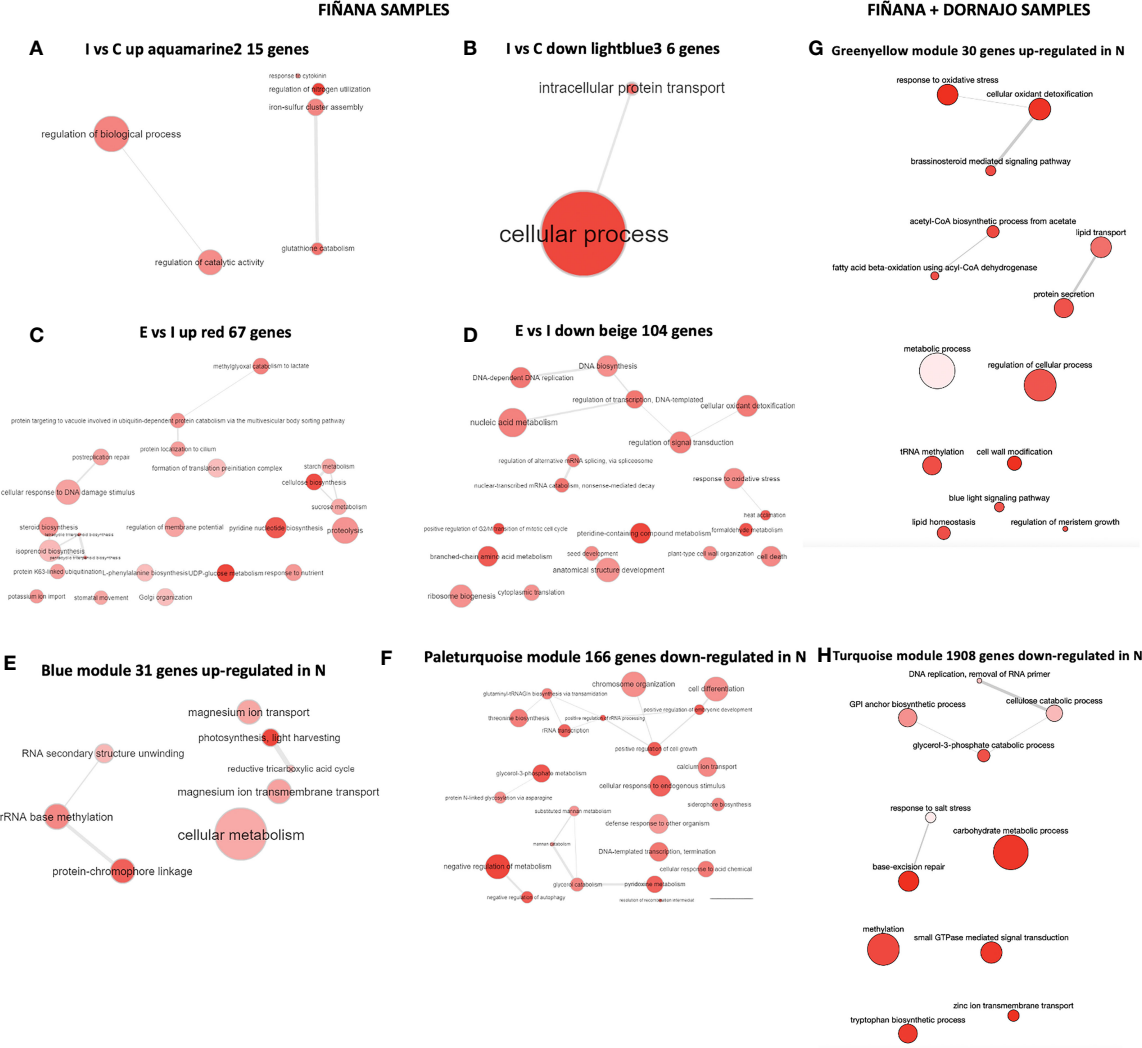
GO term enrichment results for the hub genes of the selected modules visualized using REVIGO. **(A)** Aquamarine module, I vs C up comparison (Fiñana samples). **(B)** Lightblue3 module, I vs C down comparison (Fiñana). **(C)** Red module, E vs I up comparison (Fiñana). **(D)** Beige module, E vs I down comparison (Fiñana). **(E)** Paleturquoise module, R vs N up comparison (Fiñana). **(F)** Blue module, R vs N down comparison (Fiñana). **(G)** Turquoise module, R vs N up comparison (Fiñana + Dornajo). **(H)** Greenyellow module, R vs N down comparison (Fiñana + Dornajo).

GO term enrichment analysis showed that the most abundant GO terms in all selected modules were biologically meaningful (**Figure 9**). Given the low number of DEGs, GO terms were very unspecific during I treatment, mostly related with metabolic activity among up-regulated genes and protein transport among the down-regulated (**Figures 9A, B**). Given the higher number of DEGs during E treatment, GO terms were more specific to plant drought response (**Figure 9C**). In particular, stomatal movement, potassium ion import, regulation of membrane potential, cell wall reorganization, sugar metabolism, cellular response to DNA damage stimulus, and protein ubiquitination GO terms were found among the up-regulated genes (**Figure 9C**). All these functions are related to plant response during drought stress (Kahur et al. 202, Tenhaken, 2015; Chen et al., 2021; Cobo-Simón et al., 2022, see DroughtDB Alter 2015, see Discussion). Among the down-regulated, the more prevalent GO terms were related to DNA/RNA biosynthesis and regulation, cell wall organization and anatomical structure development (**Figure 9D**), related to aerial plant growth inhibition, which can be observed during drought stress response in plants (Miao et al., 2017). Finally, the most prevalent GO terms among the up-regulated genes in N individuals were related to photosynthetic activity, cellular metabolism and RNA activity (including methylation) in both Fiñana and Fiñana+Dornajo samples, as well as cell wall modification, aerial growth and response to oxidative stress in Fiñana+Dornajo samples, all of them related to drought stress response in plants (**Figures 9E, G**, see Discussion). The down-regulated genes in N individuals were mostly related to cell division and negative regulation of metabolism in Fiñana samples (**Figure 9F**), and metabolic and methylation activity in Fiñana + Dornajo samples (**Figure 9H**, see Discussion).

#### 3.2.6 Genetic differences between responses and locations

**Figure 10:**
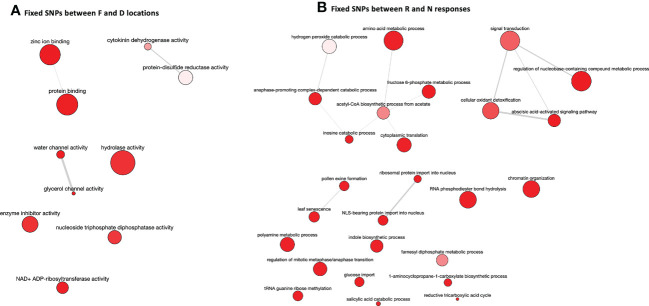
GO term enrichment results for genes containing fixed SNPs between F and D locations **(A)** and R and N responses **(B)** visualized using REVIGO.

**Figure 11:**
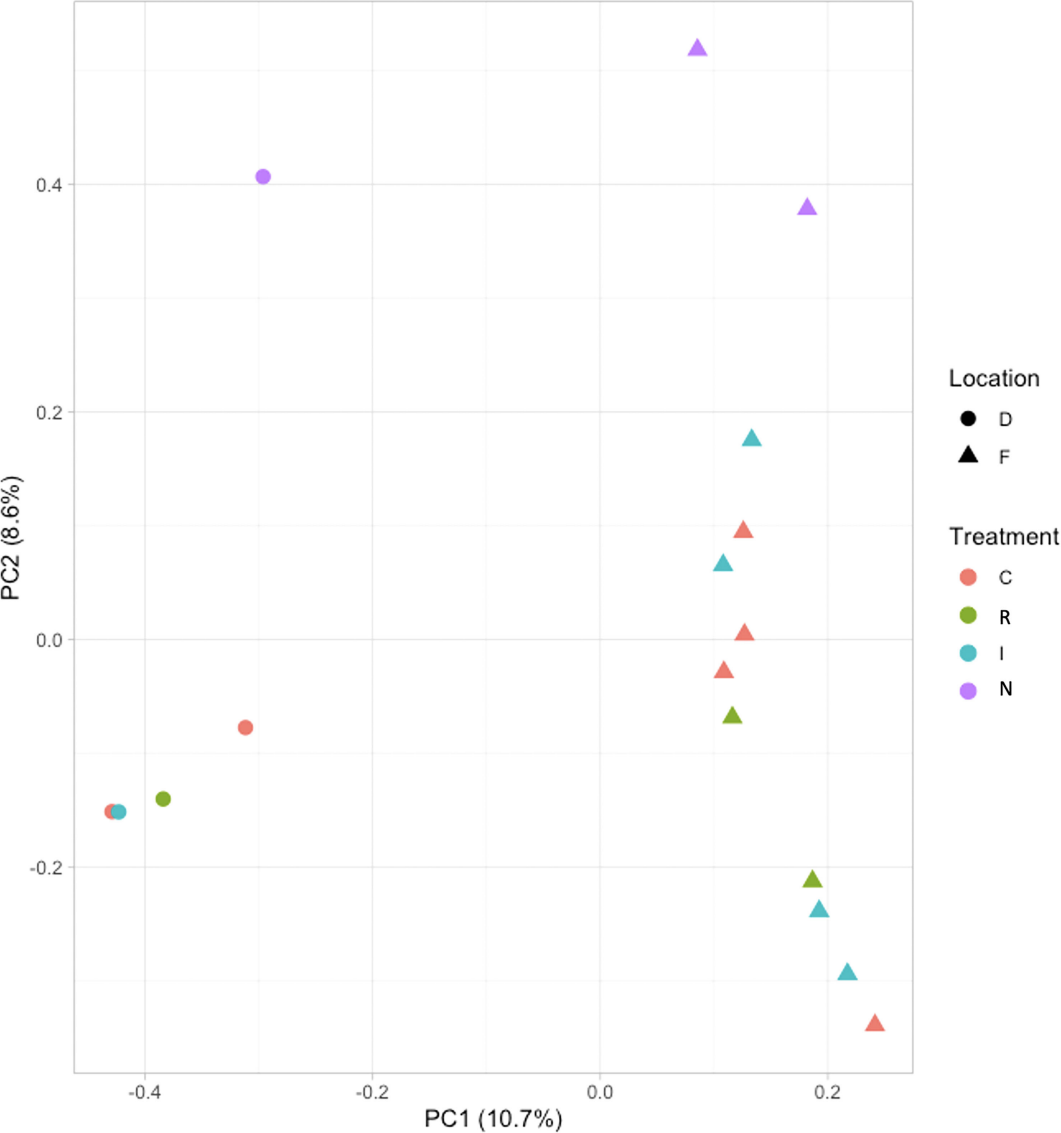
Principal component analysis (PCA) of the SNPs of the 17 samples from Fiñana and Dornajo populations.

SNP calling results, conducted in the two post-drought responses (R and N) and locations (Fiñana and Dornajo) (**Tables S12**, **S13**), were visualized by a Principal Component Analysis (PCA) (**Figure 11**). Although the number of biological replicates is not enough to draw statistically robust conclusions, principal component 1 (PC1) clearly separated the two locations, while principal component 2 (PC2) separated the N response from the rest of the individuals. These SNP-based results are clearly consistent with the PCA and the dendrogram based on gene expression differences (**Figures 4**, **5**), which supports these results. We identified 48,002 SNPs with fixed alleles between R and N samples, located in 24,829 genes, 7,392 of which were successfully annotated (**Table S13**). Four of these fixed-allele genes were found among the down-regulated DEGs between R and N (**Table 3**) and six among the up-regulated DEGs (**Table 4**). 134,924 SNPs with fixed alleles were identified between Fiñana and Dornajo populations, located in 51,654 genes, of which 15,725 were successfully annotated (**Table S12**). It was remarkable the great abundance of transposon-related genes containing the fixed SNPs between responses and locations (**Tables S12**, **S13**). In particular, *Retrovirus-related Pol poly from transposon TNT 1-94* whose function is related to the catalytic activity to produce diphosphate from DNA/RNA (Grandbastien et al., 1989). This diphosphate-related catalytic activity can be observed in the GO term enrichment results (nucleoside triphosphate-diphosphate activity, **Figure 10A**; RNA phosphodiester bond hydrolysis, **Figure 10B**). Other functions related to drought response were found among the GO terms between locations (e.g. zinc ion binding, water channel activity, **Figure 10A**) and responses (e.g. abcisic acid activated signaling pathway, cellular oxidant detoxification, glucose import, **Figure 10B**) (see DroughtDB, Alter 2015), proving that the SNP-containing genes are biologically meaningful.

**Table 3:** Overlapping contigs between the fixed SNPs between N and R individuals and the down-regulated genes in N individuals.

Contig	GO function
ABIES09_047144T3	Pectinesterase 31
ABIES09_047752T1	spermidine synthase
ABIES09_057884T2	phosphatase 2C 57
ABIES09_061593T2	glutathione peroxidase, partial

**Table 4:** Overlapping contigs between the fixed SNPs between N and R individuals and the up-regulated genes in N individuals.

Contig	GO function
ABIES09_011948T8	cannabidiolic acid synthase-like
ABIES09_014618T8	methyltransferase DDB_G0268948
ABIES09_016813T3	7-deoxyloganetin glucosyltransferase-like
ABIES09_037932T4	1-aminocyclopropane-1-carboxylate oxidase 5-like
ABIES09_041278T3	ORF124 (chloroplast)
ABIES09_065735T2	thaumatin, partial

## 4 Discussion

This work constitutes the first study in investigating the drought response and post-drought recovery in *Cedrus atlantica* from an ‘omics’ perspective (RNA-seq). Moreover, we investigate its drought resilience by comparing the eco-physiology and gene expression patterns of two contrasting post-drought recovery phenotypes (drought resilient R and drought sensitive N). This comparison is key to understand the relative importance of drought response plasticity vs genetic differences in trees’ drought resilience, whose underlying gene expression patterns are still largely unknown (Gailing et al., 2009; Neale and Kremer, 2011; Moran et al., 2017). This experimental design has been proved to be successful in identifying candidate genes of drought resilience in other relict conifers (e.g. Cobo-Simón et al., 2022). However, the main novelty of this work is the use of afforested populations as a natural experiment to investigate molecular and functional mechanisms that might contribute to rapid local adaptation to drought in conifers. To the best of our knowledge, it has never been performed in conifers.

Our study illustrates the dynamic response to drought in *C. atlantica*. Here, we describe the molecular responses that accompany the physiological response of *C. atlantica* needles to drought stress, and therefore, their gas exchange, and carbon and water balances. We hypothesized contrasting gene expression related to carbon and water balance variability in response to drought stress and causing the two contrasting post-drought recovery phenotypes in both locations. In addition, we hypothesized a rapid local adaptation in both populations. The lack of biological replicates per treatment/response prevented us from testing these hypotheses in Dornajo population. Nevertheless, we obtained enough biological replicates per population (five in Dornajo and ten in Fiñana) to draw statistically robust conclusions for a possible rapid local adaptation. In this sense, our transcriptomic results (**Figures 4**, **5**) clearly separated both populations, supporting a rapid local adaptation and our initial hypothesis. Despite the low number of samples, our SNP-based results displayed a clearly consistent pattern with the DE analysis results (**Figure 11**), separating both locations. Thus, they give support to our results and help guide future adaptative potential research in conifers.

Another remarkable finding was the higher percentage of DEGs in all pairwise comparisons when analyzing both populations together, in comparison with Fiñana samples (**Table 1**). In contrast, we found more gene modules with expression patterns of interest, related to the treatment/responses, in Fiñana samples (**Figures 8A, B**). A possible explanation might be that, despite the higher number of DEGs when analyzing both populations together, these DEGs might contain more noisy data, as a consequence of the gene expression differences between populations (**Figures 4**, **5**). This explanation is supported by the fact that the only gene modules with expression patterns of interest when analyzing both populations were found for N individuals (**Figure 8B**). Furthermore, N was the only treatment/response that clustered between populations based on gene expression (**Figures 4**, **5**), supported by the SNP-based results (**Figure 11**). In contrast, when analyzing Fiñana samples, we obtained a gene module with an expression pattern of interest per each pairwise comparison, and for both up and down-regulated DEGs (**Figure 8A**), similarly to *A. pinsapo* (Cobo-Simón et al., 2022). All these results support, again, a rapid local adaptation, as well as a molecular singularity of N individuals in *C. atlantica*.

Rapid local adaptation to increasing drought has been previously reported in other relict coniferous species (e.g. Cobo-Simón et al., 2021). A rapid local adaptation might be possible due to the retention of genetic variants for some ecologically relevant traits, hypothesized for relicts (Kawecki, 2008). Nevertheless, this hypothetical rapid local adaptation was not supported by eco-physiological measures in this work (**Figure 3**). However, differences in other eco-physiological variables and/or traits, not measured in this study, might be possible, considering the contrasting environmental conditions to which both populations are subjected in nature, without any apparent effect on fitness, and the transcriptomic and genomic differences observed in this study (**Figures 4**, **5**, **11**). In fact, morphological differences can be observed between both populations, such as leaf density or greenness, which are both higher in Dornajo individuals. Therefore, we strongly recommend to further investigate them in future studies. On the other hand, a study of the drought-response transcriptomic profile for Dornajo using, at least, three biological replicates per treatment/response is key to detect more detailed drought-related transcriptomic differences between both populations and confirm this hypothetical rapid local adaptation. Moreover, an analysis of molecular markers (e.g. SNPs) in both populations using a wider number of samples, integrating them with other omic data (e.g. metabolomic, epigenomic, proteomic) by applying multi-omic analysis, and associating them with other traits/environmental variables of interest (e.g. GWAS, GEA) would be advisable to further investigate the molecular basis of the observed drought-related gene expression and genomic differences in this work. Moreover, the metabolic pathways elucidated with these analyses will be useful to guide the search of potential phenotypic/physiological differences between these populations, and to better understand the mechanisms of rapid local adaptation in conifers. However, it is also worth noting that, although both locations were afforested using *C. atlantica* individuals from natural stands of northern Morocco, and the most probable explanation is a rapid local adaptation to drought (the afforestation project were performed during the last century, which is a short period of time for a long-lived species), we recommend to investigate these natural stands to better elucidate the cause of the genetic differences observed in F and D locations in this study. For instance, population structure or local adaptation in the populations of origin might also explain the observed differences. Notwithstanding, considering that the molecular differences found between locations in this study are drought-related, which is consistent with the contrasting precipitation rates between F and D locations, and given the lack of differences in fitness between F and D locations despite their contrasting precipitation rates, which points to a rapid local adaptation to these contrasting precipitation rates, the most plausible explanation would be a rapid local adaptation to the local environmental conditions.

Besides, we analyzed both populations together in order to examine drought-related gene expression patterns regardless the location. Contrasting gene expression significantly modulated their response to different drought intensities and their drought sensitiveness, supporting our initial hypotheses in both analyses (using only Fiñana and both populations). Nevertheless, it was remarkable the low number of DEGs during the immediate drought response (**Table 1**). These results contrasted with those obtained for the relict fir *Abies pinsapo* using a similar experimental design. They suggest a later molecular activation of the drought stress response in *C. atlantica*, compared to *A. pinsapo*, under the same drought stress conditions, supporting the wider drought stress tolerances in *C. atlantica* (Abel-Schaad et al., 2018).

N individuals from both populations, in addition to cluster together, constituted the only treatment/response clearly separated from the rest in the two PCAs (gene expression and SNP based, **Figures 4**, **11**) and the gene expression-based dendrogram (**Figure 5**) in both populations. This molecular singularity of N individuals in *Cedrus atlantica*, which is another relevant finding of this study, was also supported by the uniquely up and down expressed genes found in N individuals from Fiñana population (**Figure 8A**), and when analyzing Fiñana and Dornajo populations together (**Figure 8B**). Again, further molecular, functional, eco-physiological and morphological research in these populations (e.g. multi-omics, GWAS) using more samples would be advisable to better understand the molecular singularity of N individuals, and thus, the mechanisms of drought resilience/sensitivity in *C. atlantica*.

These results contrast, again, with those obtained for the relict fir *Abies pinsapo* using a similar experimental design (Cobo-Simón et al., 2022). There, uniquely expressed genes were found for drought resilient individuals but not for sensitive ones, pointing to different molecular and functional strategies conferring drought resilience in these conifers. Moreover, *A. pinsapo* showed a clear separation of all treatments/responses in the gene expression-based PCA and cluster dendrograms (Cobo-Simón et al., 2022).

These circum-Mediterranean conifers (*A. pinsapo* and *C. atlantica*) are both relicts, located in altitudinal ecotones and climate-change threatened due to their susceptibility to drought (Linares, 2011; Abel-Schaad et al., 2018). Hence, both of them have been experiencing a population decline over the last decades due to climate change, together with human land use effects. Consequently, they are gradually migrating upwards in the mountain ranges where they inhabit (Linares, 2011; Abel-Schaad et al., 2018). In addition, previous studies have offered evidence of a common evolutionary history in North Africa (Scaltsoyiannes et al., 1999; Qiao et al., 2007; Linares, 2011; Alba-Sánchez et al., 2018). Hence, they have been likely subjected to very similar selective pressures. For these reasons, and given the similar experimental design of this and the previous work performed in *A. pinsapo* (Cobo-Simón et al., 2022), we consider pertinent to use *A. pinsapo* results to complement the discussion of the results of this work. It is also worth noting that previous studies using genetics of circum-Mediterranean conifers reported that genetic differentiation between species seems to depend mainly on the individual biological history instead of a common vicariant phenomenon (Jaramillo-Correa et al., 2010; Liepelt et al., 2010). However, our results contradict this affirmation regarding their molecular adaptation to drought, since they show remarkably different molecular responses in *C. atlantica* and *A. pinsapo* related to their drought resilience. Although both species are considered sensitive to drought, *A. pinsapo* is extremely sensitive (Linares, 2011) whereas *C. atlantica* shows *a priori* wider tolerances (Abel-Schaad et al., 2018). Therefore, the different drought resilience-related molecular responses between *C. atlantica* and *A. pinsapo* might explain these differences in drought tolerance, shedding light on the molecular and functional mechanisms conferring drought resilience in conifers. Furthermore, the wider drought tolerances of *C. atlantica* are supported by the lack of separation between drought treatments observed in the PCAs and dendrogram (**Figures 4**, **5**, **11**) in this work, as well as the low number of DEGs during the immediate drought (**Table 1**). On the contrary, *A. pinsapo* exhibited a clear separation between treatments/responses and a higher number of DEGs (Cobo-Simón et al., 2022). A possible explanation might be that *C. atlantica* would exhibit a more constitutive gene expression pattern related to drought response and resilience than *A. pinsapo*, which would lead to its wider tolerances (Abel-Schaad et al., 2018). This possible explanation is supported by the molecular singularity of N individuals, based on both gene expression and SNPs (**Figures 4**, **5**, **8**, **11** **Tables S8**-**S13**). A constitutive expression of drought-related genes has been previously observed in drought tolerant individuals of *P. pinaster* (de María et al., 2020).

Another remarkable finding was the great prevalence of transposon-related genes among the SNPs found between both responses (R and N, **Table S12**) and the two locations (Fiñana and Dornajo, **Table S13**), which were absent among the DEGs (**Tables S4**-**S11**). These results point to a role of transposable elements (TEs) in the hypothetical rapid local adaptation and the constitutive molecular drought resilience of *C. atlantica*. TEs have been found to have a key role on genome and adaptive evolution of conifers, as an important force in shaping gene regulatory networks, their downstream metabolic and physiological outputs, and thus their phenotypes (Liu and El-Kassaby, 2019). Although the low number of samples analyzed in this work does not allow to draw robust conclusions based on SNPs, we strongly recommend to further investigate the potential role of TEs in the rapid local adaptation and constitutive drought response of *C. atlantica* in particular, and conifers in general.

### 4.1 *C. atlantica* seedlings regulate photosynthetic activity and stomatal closing during the first 24 hours of drought, consistent with an isohydric drought response

We obtained statistically significant DEGs during immediate treatment when analyzing only Fiñana, and Fiñana and Dornajo individuals together (**Table 1**). Nevertheless, given the lack of gene modules showing an expression pattern of interest when analyzing both populations (**Figure 8B**), we will focus the discussion of the *C. atlantica* seedling transcriptional profile after 24 hours of drought on Fiñana population.

Despite the low number of significantly up and down regulated genes in the two immediate drought gene modules (**Figure 8A**), they showed biologically meaningful functions (**Figure 9A, B**). *C. atlantica* is an isohydric tree regarding its physiological response to drought, characterized by its sensitivity to the atmospheric-moisture demand, which leads to a water-saving strategy by means of stomatal closure (Spinelli et al., 2011; Sanchez-Salguero et al., 2015; Lechuga et al., 2019). This stomatal closure is accomplished by increasing abscisic acid (ABA) concentrations (Moran et al., 2017). Consequently, it leads to a pronounced reduction of stomatal conductance (Gs) and limited carbon uptake by photosynthesis (A). This isohydric pattern has been observed in this work in *C. atlantica* seedlings through a progressive decrease of A and Gs values in both populations (**Figure 3**). These findings are similar to those obtained in *A. pinsapo* seedlings using the same study design (Cobo-Simón et al., 2022), whose physiological response to drought is also isohydric.

During the 24 hours of drought treatment (I), *C. atlantica* seedling transcriptional response showed consistencies with an isohydric profile. Among the hub genes, we observed several genes related to chloroplast activity and photosynthesis among the up (isoamylase 3 chloroplastic isoform X1, high chlorophyll fluorescent 107) and down-regulated genes (probable plastid-lipid-associated 8, chloroplastic, soluble inorganic pyrophosphatase 6, chloroplastic-like), including transcription factors (transcription termination factor MTERF4, chloroplastic) (**Table S4**), pointing to a regulation of photosynthetic activity.

Hub genes related with protein transport, protein kinase activity and protein phosphorylation pathways were observed among the up- (probable inactive serine threonine- kinase scy1, receptor kinase TMK1-like, GPI ethanolamine phosphate transferase 1, coatomer subunit delta, Tetratricopeptide repeat-containing domain) (**Table S4**) and down-regulated genes (leucine-rich repeat receptor-like serine threonine tyrosine-kinase, **Table S5**). Protein phosphorylation has been associated with drought-priming heat stress tolerance in plants (Zhang et al., 2020). Interestingly, protein kinases were also extremely abundant during both immediate and extended drought stress and both up and down regulated in *A. pinsapo* (Cobo-Simón et al., 2022). Protein kinases have been reported to be involved in core-stress signaling pathways related to abiotic stress, such as drought, salinity and extreme temperatures (Zhu, 2016), including ABA production and stomatal closing (Chen et al., 2021). Finally, hub genes related with transcription regulation (lncRNA, **Table S4**) and circadian clock regulation (XAP5 CIRCADIAN TIMEKEEPER, **Table S4**) were found among the up-regulated genes. Several evidence points that circadian clock contributes to plants’ ability to tolerate different types of environmental stress, and to acclimate to them (Grundy et al., 2015), including, again, ABA production and stomatal responses under water stress (Kamrani et al., 2022).

### 4.2 *C. atlantica* seedlings perform cell wall and sugar distribution remodeling, and inhibit growth and flavonoid biosynthesis after 20 days of drought

After 20 days of drought stress (E treatment), *C. atlantica* seedlings showed a higher number DEGs, pointing to a stronger drought-related stress at this stage, compared to immediate drought response, in both analyses (using Fiñana and Fiñana+Dornajo individuals) (**Table 1**). Again, given the lack of gene modules showing an expression pattern of interest when analyzing both locations together, we will focus the discussion of the gene expression profile after 20 days of drought on Fiñana population. Similar to immediate response, *C. atlantica* transcriptional profile was consistent with an isohydric drought response (**Figure 9C, D**). Hub genes related to stomatal closure, ABA transportation (NRT1 PTR family-like proteins, Guo et al., 2003 Chiba et al., 2015; acetate butyrate ligases AAE7, Dong et al., 2018) (**Table S6**), and ABA homeostasis (probable E3 ubiquitin-ligase XERICO, Ko et al., 2006) (**Table S6**) were activated. In addition, hub genes related to photosynthesis (photosystem I reaction center subunit VI-1, chloroplastic; metal transporter Nramp3-like, Lanquar et al., 2010) (**Table S7**) and metabolism (mitochondrial adenine nucleotide transporter ADNT1 and phosphoglycerate kinase, both involved in ATP production; lysosomal Pro-X carboxypeptidase, involved in proteolysis; coatomer subunit delta, involved in Golgi function) (**Table S7**) were inhibited.

In addition, cell wall-related hub genes (probable E3 ubiquitin- ligase HIP1 isoform X1, Sun et al., 2009, exocyst complex component EXO70A1 Synek et al., 2006) (**Table S6**), together with carbohydrate/sugar metabolism and transportation-related genes (malate synthase, glyoxysomal, acetate butyrate ligase AAE7, peroxisomal, Allen et al., 2011, sugar transporter 7-like, which might contribute to the uptake and recycling of cell wall sugars, Rottmann et al., 2018) (**Table S6**) were activated in *C. atlantica* seedlings after 20 days of drought. Other plant growth-related hub genes were, conversely, inhibited (probable purine permease 11, Qi and Xiong, 2013; kinesin KIN-14I, Cooper et al., 2003; beta-tubulin, Eckardt, 2006; probable strigolactone esterase DAD2, involved in plant shoot branching, Hamiaux et al., 2012; cellulose synthase E6, involved in synthesis of cell wall cellulose) (**Table S7**), including plant polyamine biosynthesis-related genes (ornithine decarboxylase-like, Illingworth and Michael, 2012; Chen et al., 2019) (**Table S7**).

Sugar transport, distribution and signaling has a critical role in plants under drought stress conditions (Kaur et al., 2021). Abiotic stress and especially drought stress-mediated injury results in reprogramming of sugar distribution across the cellular and subcellular compartments (Kaur et al., 2021). Plant cell walls are composed of carbohydrate polymers, lignin and structural proteins in variable amounts (Tenhaken, 2015). Plants exposed to drought stress reduce shoot growth while maintaining root growth to improve water intake (Miao et al., 2017), a process requiring differential cell wall synthesis and remodeling (Tenhaken, 2015). A cell wall and sugar distribution remodeling in needles, where the RNA from this work was obtained, in order to allow further growth of stressed organs (roots) to improve water uptake under drought conditions, might explain these cell wall and sugar metabolism-related genes activation, and the inhibition of growth and cell division, in aerial tissues. Metabolic pathways implied in shoot:root ratio decrease were observed in *A. pinsapo* seedling needles, related to their drought resilience as well (Cobo-Simón et al., 2022). We do not have any measurements from this work to test this hypothetical decrease in the shoot:root ratio. Besides, the time spam of this experiment and the limited size of the plants pose limitations to reliably detect it. However, we strongly recommend to test the possible role of a shoot:root ratio decrease in the drought resilience of conifers in future studies, suggested by the results of this and previous works (Cobo-Simón et al., 2022).

Other activated hub genes were related to transcription regulation under environmental stress (regulator of nonsense transcripts 1 homolog, Shi et al., 2012, heavy metal-associated isoprenylated plant 23-like, Braga de Abreu-Neto et al., 2013), including TFs (LHY isoform X1, Moenga et al., 2020) and histone demethylases (FLOWERING LOCUS D isoform X1, related to the epigenetic regulation of flowering in *A. thaliana*, Luo et al., 2015). Since *C. atlantica* is a conifer, this histone demethylase might have another function in this species, probably related to the epigenetic regulation of drought response, which would need further investigation. Epigenetic regulation-related genes were also found in *A. pinsapo* seedlings, associated to their drought resilience (Cobo-Simón et al., 2022). In addition, a gene related to alternative splicing under salt stress response in *Arabidopsis* was inhibited (serine arginine-rich splicing factor 4-like, **Table S7**) since its overexpression produces hypersensitivity of salt stress (Li Y. et al., 2021)

Other genes related to water stress response were activated (water-stress inducible 1, partial, probable carboxylesterase 15, Cao et al., 2019, linked to drought stress response in *Abies alba* Behringer et al., 2015), including, again, protein phosphorylation (receptor kinase HAIKU2, associated with drought tolerance in *Sequoia sempervirens*, de la Torre et al., 2022), similar to the immediate drought treatment.

A gene related to GABA biosynthesis was found among the down-regulated hub genes (glutamate decarboxylase-like, **Table S7**). GABA has a protective role on plants subjected to abiotic stress. It enhances drought stress tolerance by improving photosynthesis, inhibiting reactive oxygen species (ROS) generation, activating antioxidant enzymes, and regulating stomatal opening (Li et al., 2021). However, its potential role in *C. atlantica* drought response requires further investigation, given its versatile role in plants (Li et al., 2021) and its down-regulation during drought stress in this species (**Table S7**). This down-regulation might be a consequence of the inhibition of polyamine biosynthesis-related genes, previously described, since GABA can be synthesized through the polyamine metabolic pathway (Li et al., 2021).

Flavonoid metabolism-related hub genes were inhibited after 20 days of drought stress (isoflavone reductase and flavonoid 3,5 -hydroxylase, **Table S7**). It has been suggested that flavonoids protect plants against abiotic stress, and thus, their accumulation is induced rapidly upon drought (Munné-Bosch et al., 2009; Nakabayashi et al., 2014). Nevertheless, flavonoid production has been found to be inhibited in leaves during drought stress in several plants (Salekdeh et al., 2002, Pandey et al., 2010; Rani et al., 2012) including conifers (Fox et al., 2018). The pattern of down-regulation observed in our study may suggest that the plant limits the energy-intensive biosynthesis of secondary metabolites in favor of drought-related survival mechanisms.

Finally, mildew resistance locus o (Mlo) gene (MLO6), which loss-of-function confer broad-spectrum resistance to almost all known isolates of the fungal barley powdery mildew pathogen in plants (Acevedo-Garcia et al., 2014), was inhibited (**Table S7**). However, it is known that its function is not restricted to plant-powdery mildew interactions (Acevedo-Garcia et al., 2014). Therefore, a further investigation of its possible role in *C. atlantica* drought response would be advisable.

### 4.3 Drought sensitive individuals fail in metabolism and photosynthesis regulation under drought stress, and in limiting the energy-intensive biosynthesis of secondary metabolites

We hypothesized that differences in gene expression will cause the two contrasting post-drought recovery phenotypes (R and N). The identification of uniquely DEGs in drought sensitive individuals (N) was one of the main findings of this study (**Table 1**) and supports our initial hypothesis. In addition, we found gene modules showing an expected expression pattern based on our experiment and biologically meaningful functions in both analyses (using only Fiñana population, **Figure 8A**, **9E, F**; and both populations, **Figure 8B**, **9G, H**), up (**Tables S9**, **S11**) and down-regulated (**Tables S8**, **S10**). These results contrast with those obtained in *A. pinsapo* seedlings using a similar experimental design (Cobo-Simón et al., 2022), where uniquely DEGs were found for R individuals instead. These results suggest different molecular strategies conferring drought resilience in these drought sensitive and biogeographically related conifers (Scaltsoyiannes et al., 1999; Qiao et al., 2007; Linares, 2011; Alba-Sánchez et al., 2018), which would probably explain the wider drought stress tolerances in *C. atlantica* (Abel-Schaad et al., 2018).

Drought sensitive individuals from both analyses (F and F+D) showed an activation of flavonoid metabolism compared to drought resilient ones (isoflavone reductases, **Table S9**; Transparent testa 12, Debeaujon et al., 2001, **Table S11**; and a Transcription factor TT2, **Table S9**, which is involved in flavonoid metabolism when interacting with MYB TFs, Zimmermann et al., 2004). MYB TFs were found among the up-regulated genes in N individuals in both analyses as well (**Tables S9** and **S11**). These results contrast with those obtained for the extended drought response, where the flavonoid metabolism was down-regulated. Hence, they support, again, that limiting the energy-intensive biosynthesis of secondary metabolites in favor of drought-related survival mechanisms might enhance drought resilience in this species. Moreover, an activation of terpene production-related hub genes was found in N individuals from both analyses (7-deoxyloganetin glucosyltransferase-like, involved in irioid biosynthesis, Asada et al., 2013), supporting this hypothesis.

Several hub genes related to ethylene production were up-regulated in N individuals from both analyses (1-aminocyclopropane-1-carboxylate oxidase 5-like, Qin et al., 2007) (**Tables S9**, **S11**), including TFs (ethylene-responsive transcription factor 2-like, ethylene-responsive transcription factor 1A-like) (**Tables S9**, **S11**). Ethylene is a phytohormone involved in the regulation of gene expression by stress factors and by components of stress signal transduction pathways (Fujimoto et al., 2000). In addition, several hub genes related to antifungal response were activated: chitinases (basic endochitinase, partial; chitinase 2-like, class VII chitinase), peroxidases/oxidoreductases ((R)-mandelonitrile lyase-like; blue copper-like, De Rienzo et al., 2000; cannabidiolic acid synthase-like, Taura et al., 2007), thaumatin-like proteins (Ruiz-Medrano et al., 1992; de Jesús-Pires et al., 2020), beta 1,3 glucanase, Balasubramanian et al., 2012), and others (wall-associated receptor kinase-like 20, Delteil et al., 2016, zinc finger ZAT5-like, zinc finger CCCH domain-containing 17, Gupta et al., 2012; TIR NBS LRR disease resistance, McHale et al., 2006) (**Tables S9**, **S11**). These anti-fungal proteins are usually co-expressed (Balasubramanian et al., 2012) and activated in plants by ethylene and other phytohormones under several biotic and abiotic stress conditions (e.g. Kasprzewska, 2003), like wounding, osmotic pressure, cold, heavy metal stress and salt in plants (e.g. Vaghela et al., 2022). Furthermore, ethylene has been found to induce flavonoid accumulation in plants (Lewis et al., 2011). All these findings point to ethylene as an important phytohormone in the drought sensitivity of this species, probably causing a failure in limiting the energy-intensive biosynthesis of secondary metabolites.

Other TFs and transcriptional regulation-related genes were activated in both populations (lncRNA, DRE-binding 2, **Table S9**; two-component response regulator ORR21 isoform X1, probable WRKY transcription factor 4, dynein light chain 1, cytoplasmic, Stelter et al., 2007; nuclear pore complex NUP 35, **Table S11**), including one related to epigenetic regulation (methyltransferase DDB_G0268948, **Table S9**). This methyltransferase supports, again, the role of epigenetic regulation during drought stress in this species, reported in *A. pinsapo* as well (Cobo-Simón et al., 2022). In addition, an E3 ubiquitin-ligase MIEL1 was up-regulated (**Table S11**), which mediates degradation of MYB-like TFs, weakening plant defense to pathogens (Marino et al., 2013). Therefore, the expression of this gene could also explain the drought sensitiveness of these individuals.

Furthermore, detoxification proteins were found among the up-regulated genes (detoxification 40-like, Li et al., 2002) (**Tables S9**, **S11**) in N individuals, which might be a consequence of their higher levels of drought stress due to their sensitiveness to drought.

Finally, photosynthesis **(**probable 1-deoxy-D-xylulose-5-phosphate synthase 2, chloroplastic; ORF124), and metabolism-related pathways (probable fructokinase-4, Stein and Granot, 2018, cytochrome P450 82C4, mitochondrial; mitochondrial phosphate carrier 3, mitochondrial; glutamine–fructose-6-phosphate aminotransferase [isomerizing] 2 Vu et al., 2019; hydroquinone glucosyltransferase-like, glycosyl hydrolase; glycerol-3-phosphate dehydrogenase [NAD(+)] GPDHC1, cytosolic, Minic, 2008; acyl carrier 2, mitochondrial-like) were found among the up-regulated hub genes in N individuals when analyzing Fiñana and Dornajo populations (**Table S11**). Moreover, stomatal closure pathways were activated (sphingosine kinase, Coursol et al., 2005; probable phosphatase 2C 76 isoform X2, Yang et al., 2018; Hsu et al., 2021) (**Table S11**). It is worth noting that a phosphatase 2C was related to drought resilience in *A. pinsapo* and was also up-regulated under drought stress conditions in *Pinus halepensis* (Fox et al., 2018). These findings suggest that drought sensitivity in *C. atlantica* might be caused by failing in down-regulating photosynthesis and metabolism under drought stress conditions, which will negatively affect the whole plant carbon status.

One of the main objectives of this work was the identification of candidate genes mechanistically involved in the drought resilience of *C. atlantica*. However, our findings provide a set of drought-sensitiveness candidate genes instead, since we found uniquely up and down regulated genes in non-resilient individuals. These drought-sensitiveness candidate genes are key for the design of conservation and management strategies, as well as breeding programs. To ensure their success, it is not only necessary to know the genes conferring tolerance, but those that we aim to avoid, that is, those conferring sensitivity.

### 4.4 Genetic differences between responses and locations point to a role of transposable elements in the local adaptation and drought resilience of *C. atlantica*

Given that both post-drought responses were subjected to the same drought treatment, we hypothesized SNPs as polymorphisms associated to their differentially expressed genes, and thus, related to their drought resilience. In addition, we hypothesized a drought-related rapid local adaptation in both *C. atlantica* locations given the lack of differences in their fitness despite the contrasting precipitation rates. Both hypotheses were supported by our results, showing genomic differences between responses and locations (**Figure 11**).

The lack of clear gene expression differences between treatments and responses, with the exception of N individuals (**Figures 4**, **5**), in contrast with *A. pinsapo* results which showed a clear separation of all treatment/responses (Cobo-Simón et al., 2022), led us to hypothesize a more constitutive gene expression related to drought in *C. atlantica*, compared to *A. pinsapo*. This drought-related constitutive gene expression might lead to its wider drought tolerances (Abel-Schaad et al., 2018). The lack of genomic differences between treatments and responses, with the exception, again, of the molecular singularity of N individuals, based on the SNP results (**Figure 11**) supports this hypothesized constitutive gene expression conferring drought resilience on this species. In addition, the genomic differences between locations (**Figure 11**), consistent with their gene expression patterns (**Figure 4**, **5**), support our hypothesis of a rapid local adaptation to the contrasting precipitation rates. This hypothetical rapid local adaptation might have been favored by this hypothesized drought-related constitutive genetic diversity. Evidence of local adaptation to drought has been found in other relict conifers (*A. pinsapo*, Cobo-Simón et al., 2021). Despite the low number of samples used in the SNP calling analysis, which prevents us to draw statistically robust conclusions based on SNPs, these findings point to enough adaptive potential of this species under a climate change scenario, although further investigation is needed to fully validate it.

As expected, few SNP genes were found among the DEGs (**Tables 3**, **4**), since most of the causative variation of this DEGs is not located in the transcriptome, but in regulatory regions of the genome, both in *cis* and in *trans*. These results are similar to those obtained for *A. pinsapo* (Cobo-Simón et al., 2022). The annotation and GO term enrichment analysis of the SNP-related genes between responses (**Table S12**) and locations (**Table S13**) showed similar functions to the DEGs (**Figure 10**, **Tables S4**-**S11**). Nevertheless, the abundance of transposon-related genes in both comparisons, which were absent on the DEGs, was remarkable. These results point to a role of TEs in the local adaptation and the constitutive molecular response conferring drought resilience of *C. atlantica*.

TEs have been found to have a key role on genome and adaptive evolution of conifers, as an important force in shaping gene regulatory networks, their downstream metabolic and physiological outputs, and thus their phenotypes (Liu and El-Kassaby, 2019). In fact, the large genome size of conifers seems to result from the slow and steady accumulation of a diverse set of long-terminal repeat TEs, possibly due to a lack of an efficient elimination mechanism (Nystedt et al., 2013). TEs are DNA fragments that can move and amplify their copy number within a host genome (Casacuberta and González, 2013). As a result, they are a major and powerful source of genomic mutations, causing the evolution of new genes and their functionalities and generating novel regulatory sequences, such as promoters and enhancers (Liu and El-Kassaby, 2019). These *in cis* regulatory modifications change the regulatory and/or epigenetic environment, leading to modified gene expression (Mita and Boeke, 2016). Furthermore, TEs could generate genetic variation in response to environmental and genomic perturbations, which could facilitate adaptation (Casacuberta and González, 2013). Hence, it has been suggested that TEs may lead to adaptation to novel habitats in the face of limited genetic variation, such as invasive species (the so-called genetic paradox of invasive species, Stapley et al., 2015), which can also explain the local adaptation of relicts, such as *C. atlantica.* Hence, we strongly recommend to further investigate the potential role of TEs in rapid local adaptation and drought resilience of *C. atlantica* in particular, and conifers in general.

### 4.5 Future directions

Here we offer the first genomic resources for the climate change threatened relict *C. atlantica*, including its first *de novo* transcriptome assembly, drought-related transcriptomic profile and candidate genes of drought sensitiveness. Our results contrast with those obtained in a similar experimental design in the relict and biogeographically related fir *A. pinsapo* (Cobo-Simón et al., 2022), where uniquely expressed genes were found for drought resilient individuals instead. This finding is remarkable, specially from an evolutionary perspective, since it suggests two different molecular strategies to the same abiotic stress in both conifers (drought), which would probably explain their different drought tolerances. In addition, our results suggest a constitutive molecular drought response conferring drought resilience in *C. atlantica* and a rapid local adaptation to drought in the two studied populations. TEs might play a key role in this rapid local adaptation and its standing genetic variation related to drought resilience, hypothesized for relict species (Kawecki, 2008). Thus, we recommend further research using a statistically robust number of biological replicates per treatment/response in Dornajo population to elucidate potential drought-related molecular and functional differences between populations. Moreover, we recommend further validation of this probable local adaptation suggested by our results, and the potential role of TEs, by using a wider sample size in both locations, by integrating multi-omic data (e.g. epigenomic, metabolomic) with other traits/environmental variables of interest (e.g. GWAS, GEA) and by studying the *C. atlantica* natural stands from northern Morocco.

Our findings, especially when complemented with those from *A. pinsapo* (Cobo-Simón et al., 2022), shed light on the adaptive potential of trees in general, and relict conifers in particular, to the projected increasing drought caused by current climate change, which is particularly concerning in Mediterranean and other semi-arid climates. This information will allow us better predict their future perspectives facing current global warming.

Finally, our results offer a diversity of drought-related genomic resources, including candidate genes of drought sensitiveness, which will help guide the design of conservation strategies and breeding programs for this climate-change threatened and drought-sensitive conifer. Furthermore, they can be used to advance future drought-resilience research, such as comparative genomic studies and functional transfer across species, which will also shed light on trees’ molecular adaptive potential to current climate change.

## Data availability statement

The datasets presented in this study can be found in online repositories. The names of the repository/repositories and accession number(s) can be found below: https://www.ncbi.nlm.nih.gov/, PRJNA668002.

## Author contributions

IC-S wrote the manuscript. FG and JL conceived the idea. JL performed the field sampling. BM-C performed the RNA extraction. JS carried out the drought experiment. JL performed the eco-physiological analyses. JM, JG-G, AE-C, MD and TA carried out the *de novo* transcriptome sequencing and annotation. IC-S carried out the rest of bioinformatic analyses. ICS- conducted the results discussion. All authors contributed to the article and approved the submitted version.
