# Supplementary material for: De novo transcriptome sequencing and gene co-expression reveal a genomic basis for drought sensitivity and evidence of a rapid local adaptation on Atlas cedar (Cedrus atlantica)
Source: Front Plant Sci. 2023 Apr 19;14:1116863. doi: 10.3389/fpls.2023.1116863 (PMC10155838; doi:10.3389/fpls.2023.1116863)
Supplement: Supplementary file 1 [file DataSheet_1.docx]

**Supplementary figures**

**
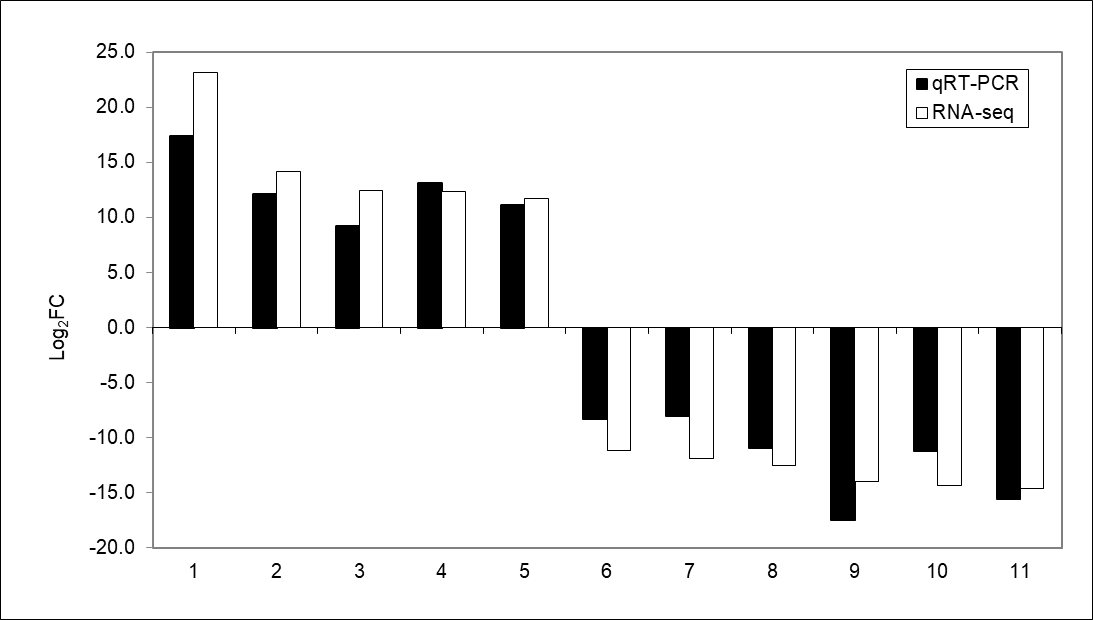
**

**Fig S1.** Correlation between the gene expression ratios obtained from RNA-Seq data and qRT-PCR. The expression ratio (Log2FC) change was calculated by the 2-ΔΔCT method. Investigated genes were listed in Table SX, including 1) 012604T8; 2) 011612T4; 3) 007640T11; 4) 030082T4; 5) 027847T2; 6) 027095T3; 7) 017209T4 ; 8) 013689T4 ; 9) 012753T3 ; 10) 091466T1; 11) 041227T1.

**Supplementary tables**

**Table S1.** Primers used for qRT-PCR analysis.

|  | **Gene code** | **Gene function**  **(putative)** | **Treatment/**  **Response*** | **log2FC**  **(RNA-seq)** | **F primer** | **R primer** |
| --- | --- | --- | --- | --- | --- | --- |
| **1** | 012604T8 | disease resistance RPS2 | R vs N | 23,17 | GTTTGTTCCAGATGCCGCTC | ATGGCCACCGTTAGTACTGC |
| **2** | 011612T4 | lncRNA | I vs C | 14,15 | TAAGCCCCGACAATCACAGG | GCCATCCTTTGCAGACTTCG |
| **3** | 007640T11 | isoamylase 3 | I vs C | 12,48 | ACTGCATCGCCCAATTGAGA | TGCCGCAACCAACTAAGAGT |
| **4** | 030082T4 | exocyst complex component EXO70A1 | E vs I | 12,36 | CCATCAACAGACAACCCCGA | AGGGTTCGGTTGAGCGAAAT |
| **5** | 027847T2 | sugar transport 7-like | E vs I | 11,72 | TCCCACACCATGCAGAGAAC | TCGGATCCCACCTTTTGAGC |
| **6** | 027095T3 | soluble inorganic pyrophosphatase 6 | I vs C | -11,21 | CCTGTAGAGAGCCCTGCATG | GCAATGCCCTTTTCCTCAGC |
| **7** | 017209T4 | DNA damage-binding 1 | I vs C | -11,85 | TTGCGGTCAGACAACTCTCC | CACCCCGATCTGTGCTTCTT |
| **8** | 013689T4 | LRR-RL serine threonine tyrosine- kinase | I vs C | -12,54 | CCTCCGTCTGTCCAAAAGCT | TAGAGGTGGGTCAGGGGAAG |
| **9** | 012753T3 | plastid-lipid-associated 8 | I vs C | -13,97 | CCCAGCCATTTCCTCTCCAT | CTGAGTGCATCGATGAGCCT |
| **10** | 091466T1 | isoflavone reductase | R vs N | -14,38 | GAAGAAACAATGGCGGCCTG | ATAGAGGCGGCCACAATAGC |
| **11** | 041227T1 | transcription factor MYB114-like | R vs N | -14,60 | GCAAAGAGGGCTGACAGACT | TAGCCCCGTGATTGATGCTT |

* C= Control, E= Extended-Drought, I= Immediate, N= Non-Resilient, R= Drought-Resilient

**Table S3.** Number of raw and clean reads (before and after quality control filtering) per sample in millions. The number on the sample name is the individual ID. The letter placed afterwards stands for the location (F for Fiñana, D for Dornajo). Finally, the last letter stands for the treatment/response (C for control, I for immediate, R for drought resilient and N for non-resilient).

| **Sample** | **Raw reads (millions)** | **Clean reads (millions)** |
| --- | --- | --- |
| 3FC (3.11) | 34.12 | 30.13 |
| 7FC (3.23) | 34.48 | 29.22 |
| 8FC (3.35) | 32.79 | 28.84 |
| 10FC (3.47) | 37.24 | 32.86 |
| 13FI (3.107) | 40.07 | 35.26 |
| 18FI (3.119) | 36.07 | 14.38 |
| 20FI (3.131) | 35.41 | 31.64 |
| 21FI (3.143) | 32.82 | 28.77 |
| 28FR (3.40) | 40.26 | 35.15 |
| 30FR (6.100) | 23.37 | 14.32 |
| 27FN (3.28) | 39.73 | 34.30 |
| 31FN | 23.40 | 15.58 |
| 34DC (6.124) | 24.61 | 21.08 |
| 36DC (6.136) | 23.03 | 19.56 |
| 41DI (6.2) | 25.85 | 21.63 |
| 51DR (8.26) | 22.70 | 17.82 |
| 47DN (6.14) | 22.99 | 19.06 |
| **Total** | **528.94** | **429.60** |

**Appendix 1.** ANOVA results of net photosynthesis (A) and stomatal conductance (Gs) monitored in controls (C), immediate drought (I, after 24 hours of drought treatment), and extended drought (E, after 20 days of drought treatment).The E group was divided into drought resilient seedlings (R) and non-resilient seedlings (N), based on recovery capacity after the extended drought. Groups are noted by the treatment/response (C, I, E; R, N) and population codes (F, D).


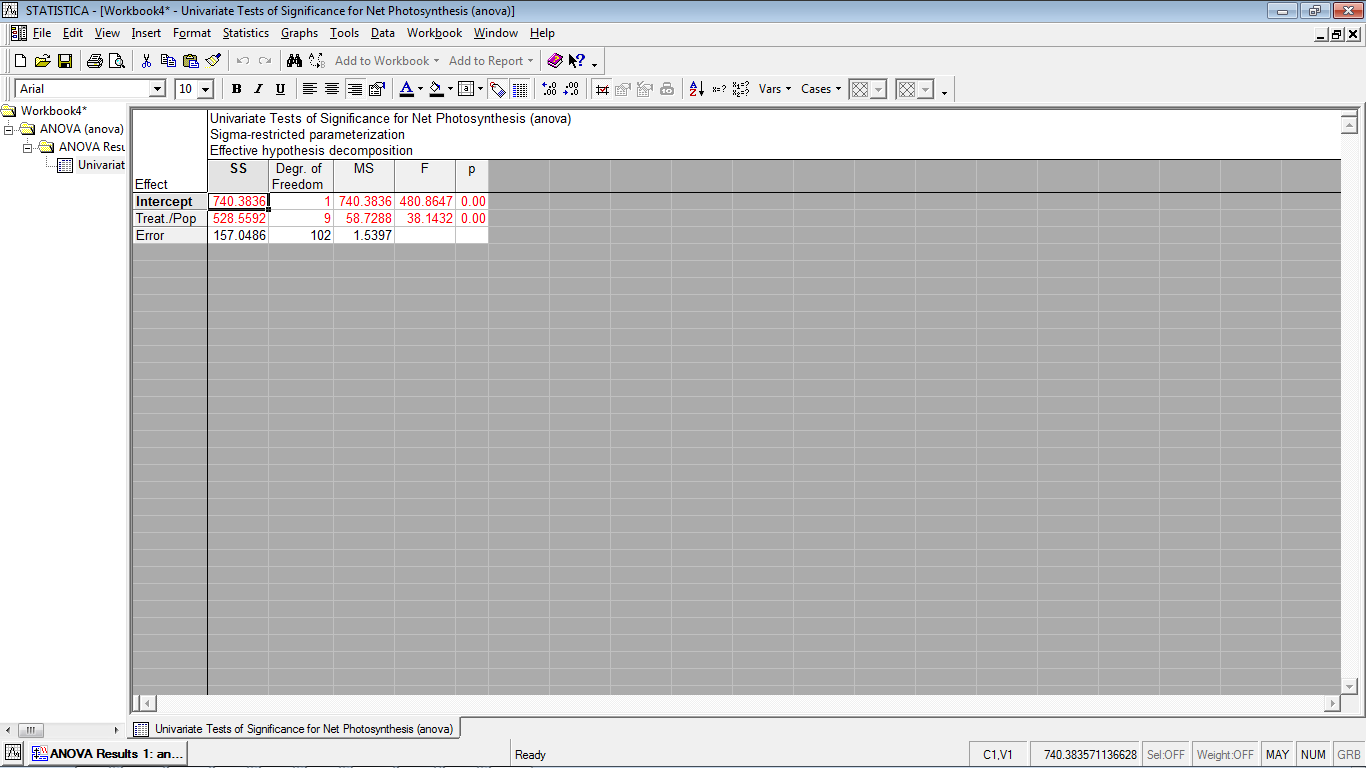


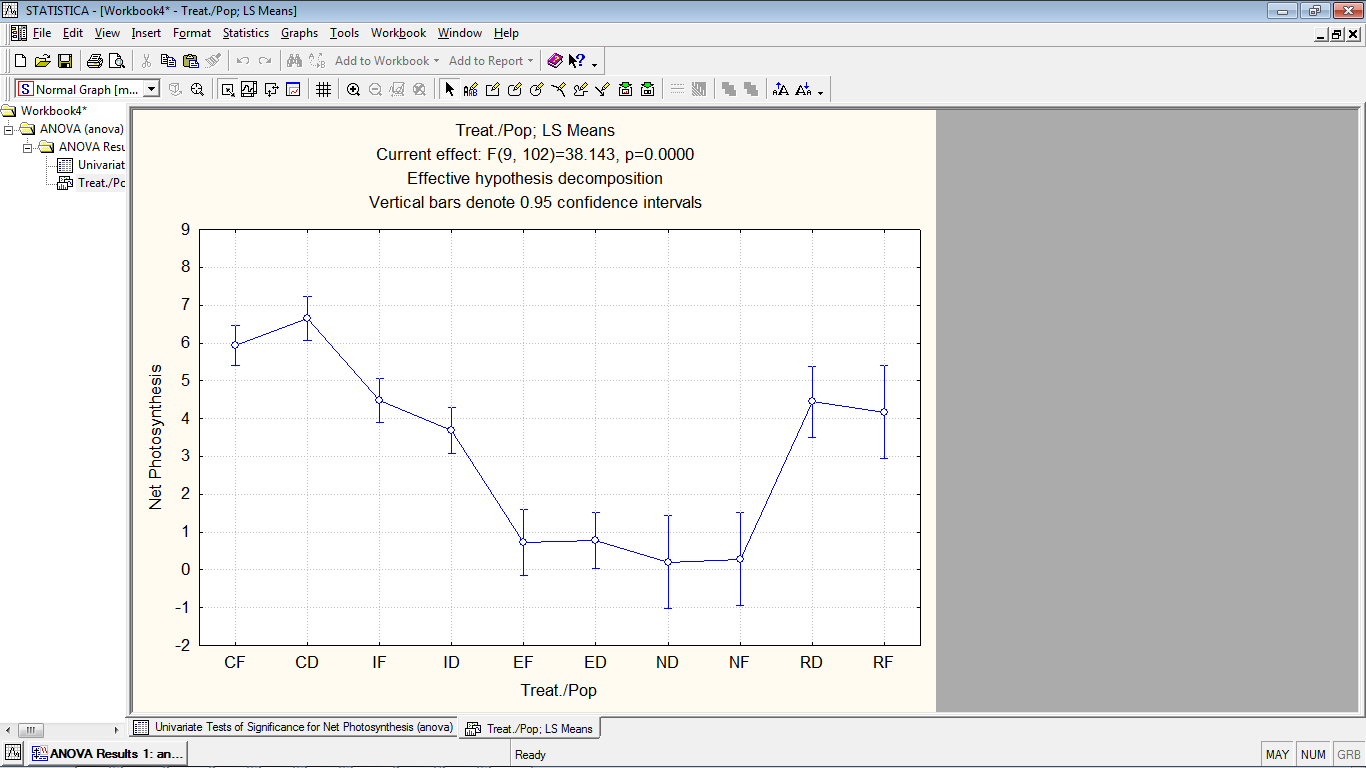


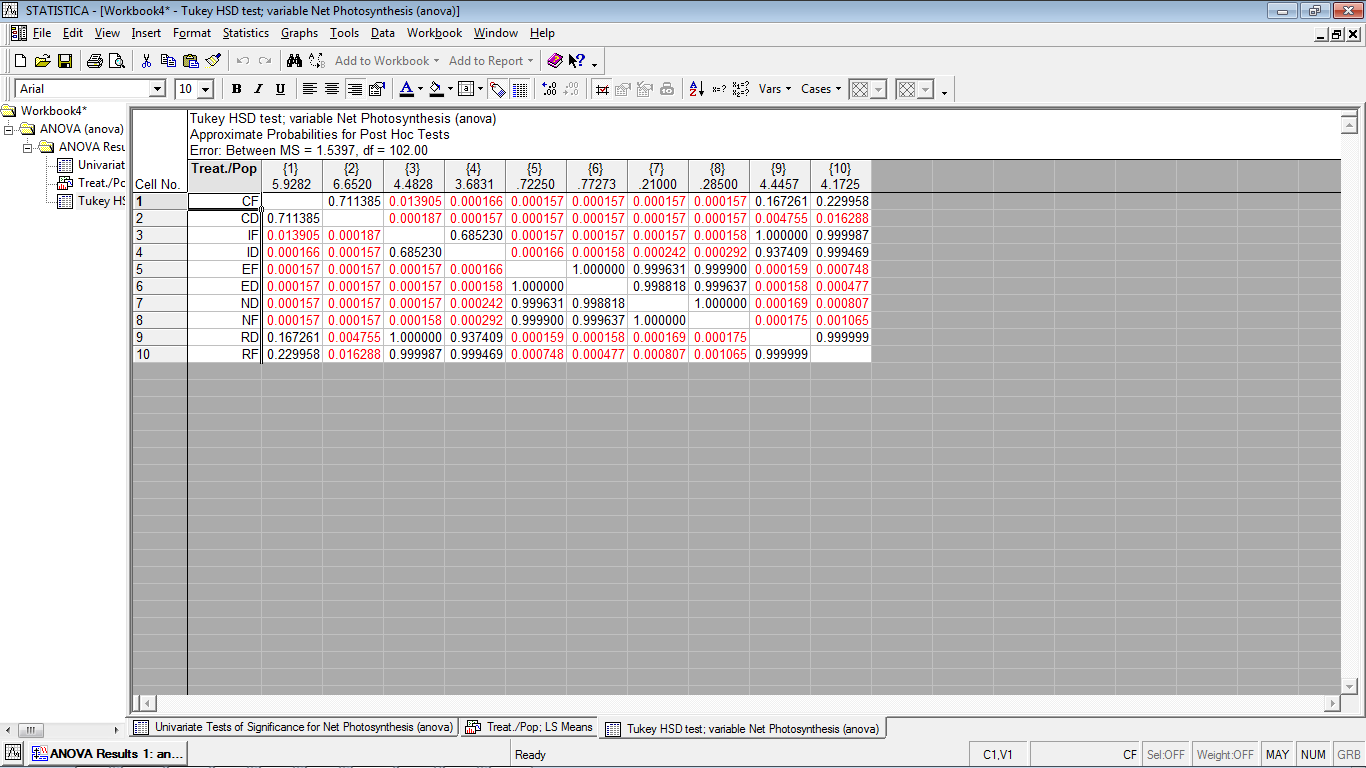


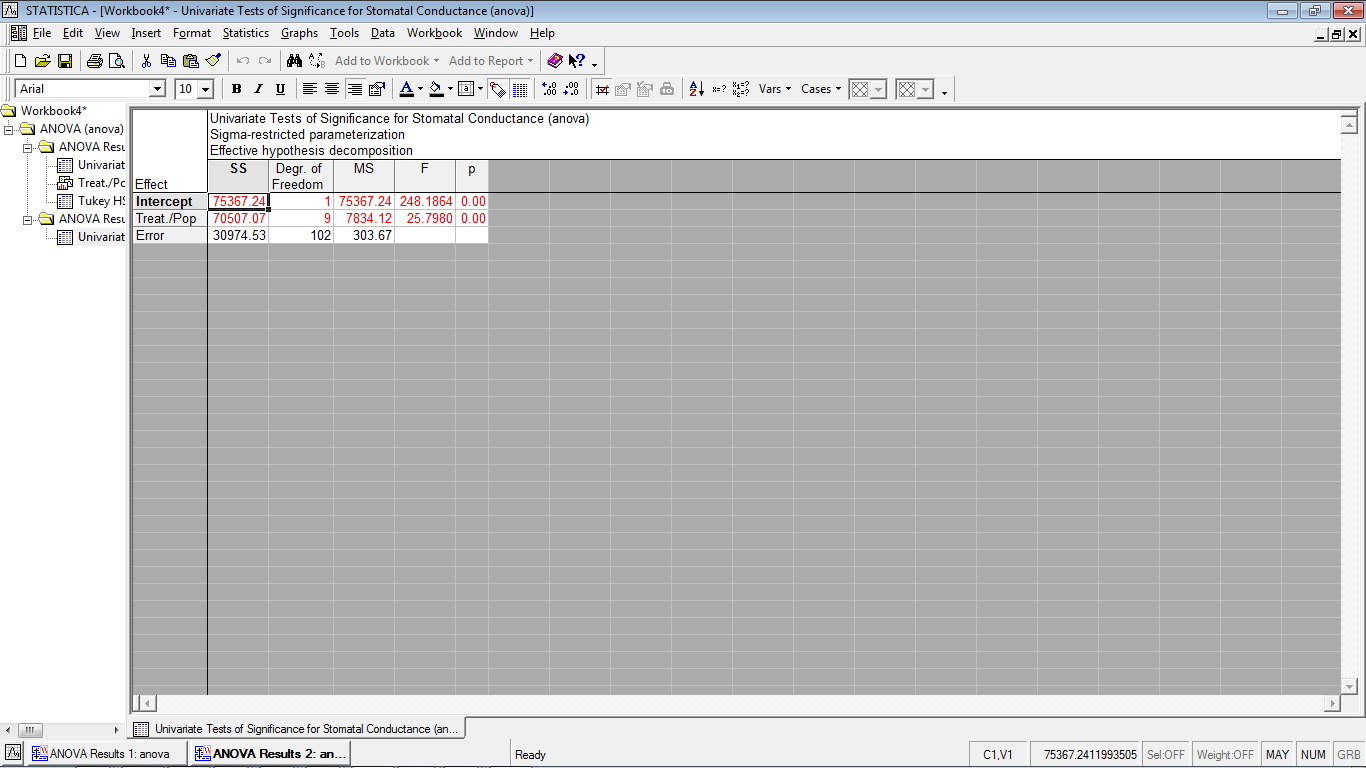


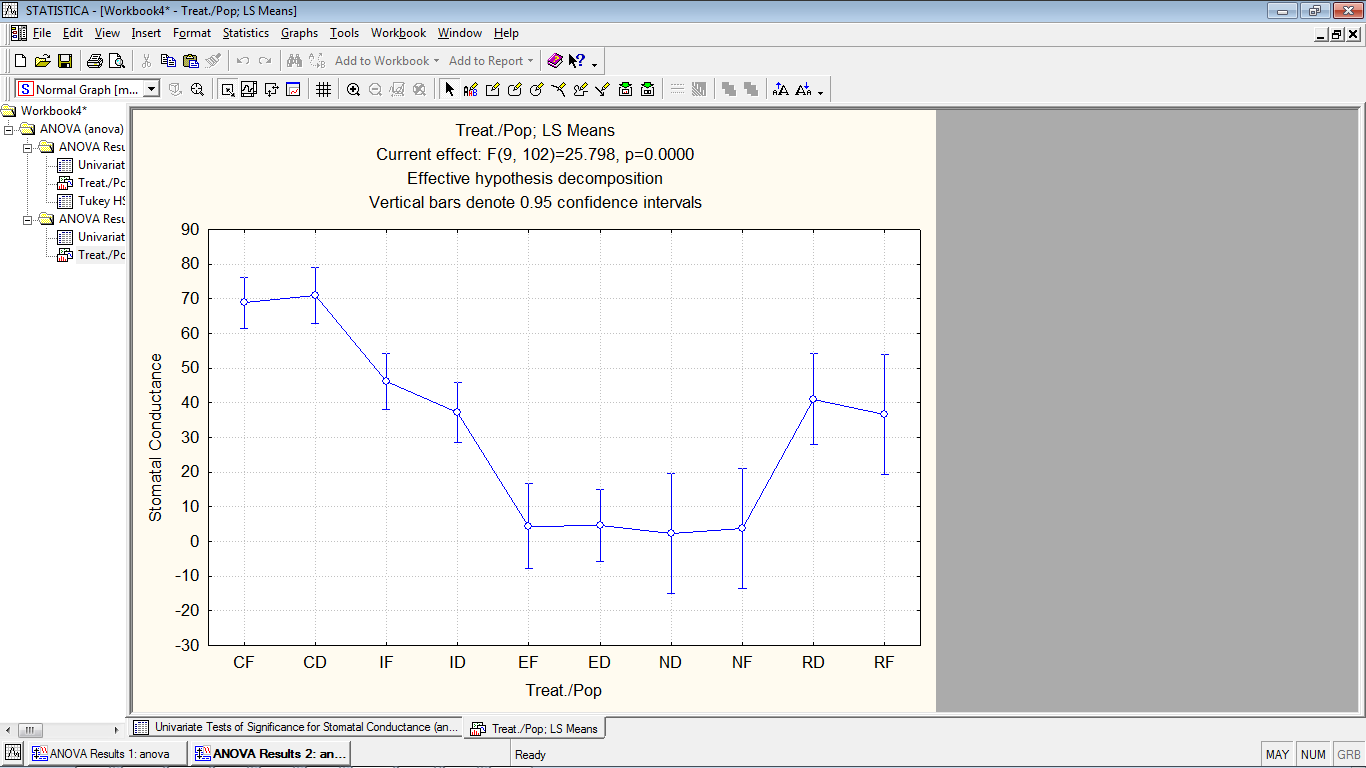


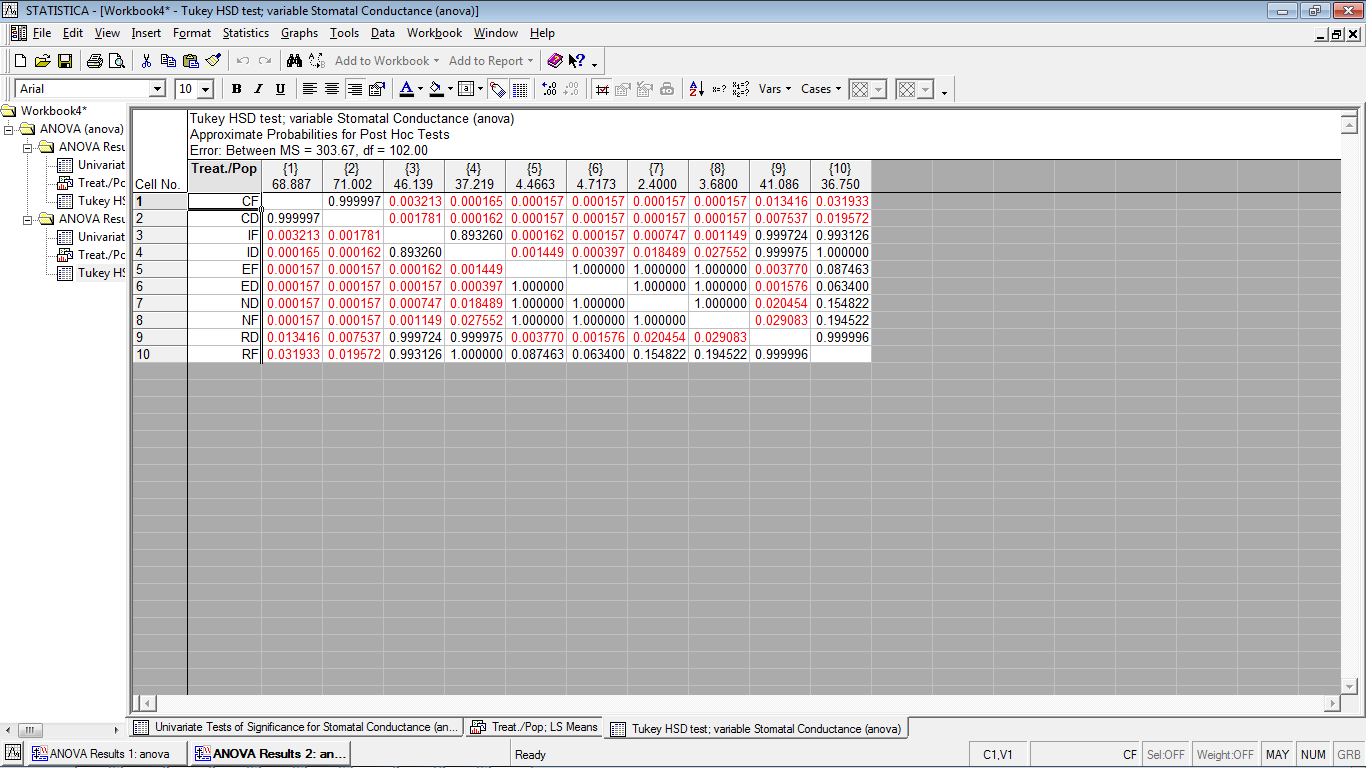


**Appendix 2: WGCNA code**

# Display the current working directory

getwd()

# If necessary, change the path below to the directory where the data files are stored.

# "." means current directory. On Windows use a forward slash / instead of the usual \.

#install.packages("BiocManager")

#BiocManager::install("WGCNA")

# Load the WGCNA package

library(WGCNA)

# The following setting is important, do not omit.

options(stringsAsFactors = FALSE)

#Read in the data set containing the read counts

counts<-read.csv("allcounts.csv", header=T, sep=",")

#normatization

#BiocManager::install("edgeR")

library(edgeR)

countsnorm<-voom(counts)

dim(countsnorm)

# get genes with large variation across samples using variance, The variance is a numerical measure of how the data values is dispersed around the mean.

# https://labs.genetics.ucla.edu/horvath/CoexpressionNetwork/Rpackages/WGCNA/faq.html

countsnorm <- as.data.frame(countsnorm)

countsnorm$variance <- as.numeric(apply(countsnorm,1,var))

head(countsnorm)

summary(countsnorm$variance) #3rd quartil 2.24391

library(tidyverse)

countsnorm.largeVar<-

countsnorm %>%

filter(variance>2.24391) %>% # dispersed one reads arround the mean

dplyr::select(-variance)

head(countsnorm.largeVar)

rownames(countsnorm.largeVar) <- rownames(countsnorm[countsnorm$variance >2.24391,])

head(countsnorm.largeVar)

save(countsnorm.largeVar, file = "countsnormcedroreduced.largeVar.Rdata")

write.csv(countsnorm.largeVar,file="countsnorm.largeVar.csv")

ID<-read.csv("countsnorm.largeVar.csv")

write.table(IDs,file="AllID.txt")

# Display the current working directory

getwd()

# If necessary, change the path below to the directory where the data files are stored.

# "." means current directory. On Windows use a forward slash / instead of the usual \.

# Load the WGCNA package

library(WGCNA)

# The following setting is important, do not omit.

options(stringsAsFactors = FALSE)

#Read in the female liver data set

countsnorm.largeVar<-read.csv("countsnorm.largeVar.csv", header=T, sep=",")

# transform expression data

head(countsnorm.largeVar[,1:6])

countsnorm.largeVar.t <- t(countsnorm.largeVar)

# check sample quality, this is to see whether there are many missing entry for certain gene, also check variance

gsg1 = goodSamplesGenes(countsnorm.largeVar.t, verbose = 3)

head(gsg1)

gsg1$allOK

# cluster samples to look for outliers based on gene expression data

# hclust 1stly calculate pairwise dissimilarity between each pair of observations, the observations that are most similar to each other are fused, then the next most similar observations... For clusters with multiple observations, use linkage to quantitate their dissimlarity, including complete, single, average, and centroid linkage.

sampleTREE.CR <- hclust(dist(countsnorm.largeVar.t), method = "average") # average is the mean intercluster dissmilarity

sizeGrWindow(12,9)

pdf(file = "Plots/sampleClustering.pdf", width = 12, height = 9)

par(cex = 0.6);

par(mar = c(0,4,2,0))

plot(sampleTREE.CR, main = "Sample clustering to detect outliers", sub="", xlab="", cex.lab = 1.5, cex.axis = 1.5, cex.main = 2)

# Plot a line to show the cut

abline(h = 15, col = "red")

# Determine cluster under the line

clust = cutreeStatic(sampleTREE.CR, cutHeight = 15, minSize = 10)

table(clust)

# clust 1 contains the samples we want to keep.

#2nd day step by step

# Display the current working directory

getwd()

# If necessary, change the path below to the directory where the data files are stored.

# "." means current directory. On Windows use a forward slash / instead of the usual \.

# Load the WGCNA package

library(WGCNA)

# The following setting is important, do not omit.

options(stringsAsFactors = FALSE);

# Allow multi-threading within WGCNA. This helps speed up certain calculations.

# At present this call is necessary for the code to work.

# Any error here may be ignored but you may want to update WGCNA if you see one.

# Caution: skip this line if you run RStudio or other third-party R environments.

# See note above.

# enableWGCNAThreads()

# Load the data saved in the first part

#This step is the bedrock of all network analyses using the WGCNA methodology.

#We present three different ways of constructing a network and identifying modules:

# a. Using a convenient 1-step network construction and module detection function, suitable for users wishing to arrive at the result with minimum effort

# Choose a set of soft-thresholding powers

powers = c(c(1:10), seq(from = 12, to=20, by=2))

# Call the network topology analysis function

sft = pickSoftThreshold(countsnorm.largeVar.t, powerVector = powers, verbose = 5, networkType = "signed")

# Plot the results:

sizeGrWindow(9, 5)

par(mfrow = c(1,2));

cex1 = 0.9;

# Scale-free topology fit index as a function of the soft-thresholding power

plot(sft$fitIndices[,1], -sign(sft$fitIndices[,3])*sft$fitIndices[,2],

xlab="Soft Threshold (power)",ylab="Scale Free Topology Model Fit,signed R^2",type="n",

main = paste("Scale independence"));

text(sft$fitIndices[,1], -sign(sft$fitIndices[,3])*sft$fitIndices[,2],

labels=powers,cex=cex1,col="red");

# this line corresponds to using an R^2 cut-off of h

abline(h=0.90,col="red")

# Mean connectivity as a function of the soft-thresholding power

plot(sft$fitIndices[,1], sft$fitIndices[,5],

xlab="Soft Threshold (power)",ylab="Mean Connectivity", type="n",

main = paste("Mean connectivity"))

text(sft$fitIndices[,1], sft$fitIndices[,5], labels=powers, cex=cex1,col="red")

#we chose 14 as soft power since it was the first value that touches the red line.

# A signed co-expression network was constructed, in which modules correspond to positively correlated genes, since it is more accurate for this work

softPower = 14;

adjacency = adjacency(countsnorm.largeVar.t, power = softPower, type="signed");

# Turn adjacency into topological overlap

TOM = TOMsimilarity(adjacency);

dissTOM = 1-TOM

rm(TOM)

geneTree = hclust(as.dist(dissTOM), method = "average");

# Plot the resulting clustering tree (dendrogram)

sizeGrWindow(12,9)

#pdf(file = "PlotsdendroTom.pdf", width = 12, height = 9)

plot(geneTree, xlab="", sub="", main = "Gene clustering on TOM-based dissimilarity",

labels = FALSE, hang = 0.04);

#dev.off()

# We like large modules, so we set the minimum module size relatively high:

minModuleSize = 30;

# Module identification using dynamic tree cut:

dynamicMods = cutreeDynamic(dendro = geneTree, distM = dissTOM,

deepSplit = 2, pamRespectsDendro = FALSE,

minClusterSize = minModuleSize);

table(dynamicMods)

# Convert numeric lables into colors

dynamicColors = labels2colors(dynamicMods)

table(dynamicColors)

# Plot the dendrogram and colors underneath

sizeGrWindow(8,6)

#pdf(file = "PlotsdendroTom.pdf", width = 8, height = 6)

plotDendroAndColors(geneTree, dynamicColors, "Dynamic Tree Cut",

dendroLabels = FALSE, hang = 0.03,

addGuide = TRUE, guideHang = 0.05,

main = "Gene dendrogram and module colors")

# Calculate eigengenes

MEList = moduleEigengenes(countsnorm.largeVar.t, colors = dynamicColors)

MEs = MEList$eigengenes

# Calculate dissimilarity of module eigengenes

MEDiss = 1-cor(MEs);

# Cluster module eigengenes

METree = hclust(as.dist(MEDiss), method = "average");

# Plot the result

sizeGrWindow(7, 6)

plot(METree, main = "Clustering of module eigengenes",

xlab = "", sub = "")

MEDissThres = 0.25

# Plot the cut line into the dendrogram

abline(h=MEDissThres, col = "red")

# Call an automatic merging function

merge = mergeCloseModules(countsnorm.largeVar.t, dynamicColors, cutHeight = MEDissThres, verbose = 3)

# The merged module colors

mergedColors = merge$colors;

# Eigengenes of the new merged modules:

mergedMEs = merge$newMEs;

sizeGrWindow(12, 9)

#pdf(file = "Plots/geneDendro-3.pdf", wi = 9, he = 6)

plotDendroAndColors(geneTree, cbind(dynamicColors, mergedColors),

c("Dynamic Tree Cut", "Merged dynamic"),

dendroLabels = FALSE, hang = 0.03,

addGuide = TRUE, guideHang = 0.05)

#dev.off()

# Rename to moduleColors

moduleColors = mergedColors

# Construct numerical labels corresponding to the colors

colorOrder = c("grey", standardColors(50));

moduleLabels = match(moduleColors, colorOrder)-1;

MEs = mergedMEs;

moduleColors

unique(moduleColors)

# Save module colors and labels for use in subsequent parts

save(MEs, moduleLabels, moduleColors, geneTree, file = "Cedroreducedallcountshighvariance-02-networkConstruction-stepByStep.RData")

lnames = load(file = "Cedroreducedallcountshighvariance-02-networkConstruction-auto.RData")

# Rename to moduleColors

moduleColors = mergedColors

# Construct numerical labels corresponding to the colors

colorOrder = c("grey", standardColors(50));

moduleLabels = match(moduleColors, colorOrder)-1;

MEs = mergedMEs;

moduleColors

unique(moduleColors)

# Save module colors and labels for use in subsequent parts

save(MEs, moduleLabels, moduleColors, geneTree, file = "Cedroreducedallcountshighvariance-02-networkConstruction-stepByStep.RData")

lnames = load(file = "Cedroreducedallcountshighvariance-02-networkConstruction-auto.RData")

write.table(moduleColors, file="Modulecolorsmergedsigned.txt", sep="")
